# *O*-GlcNAcylation of NONO regulates paraspeckle component assembly and contributes to colon cancer cell proliferation

**DOI:** 10.1038/s41420-025-02405-z

**Published:** 2025-05-13

**Authors:** Yeolhoe Kim, Kyung-Tae Lee, Han Byeol Kim, Hyeryeon Jung, Jeong Yeon Ko, Tae Hyun Kweon, Hari Chandana Yadavalli, Junghwa Seo, Suena Ji, Yun Ju Kim, Donghyuk Shin, Seong Wook Yang, Myeong Min Lee, Jin Won Cho, Eugene C. Yi, Jin-Wu Nam, Won Ho Yang

**Affiliations:** 1 Department of Systems Biology, College of Life Science and Biotechnology, Yonsei University, Seoul, Republic of Korea; 2 Department of Life Sciences, College of Natural Science, Hanyang University, Seoul, Republic of Korea; 3 Department of Molecular Medicine and Biopharmaceutical Sciences, School of Convergence Science and Technology and College of Medicine or College of Pharmacy, Seoul National University, Seoul, Republic of Korea; 4 Glycosylation Network Research Center, Yonsei University, Seoul, Republic of Korea

**Keywords:** Glycobiology, Glycosylation, Cancer metabolism

## Abstract

Non-POU domain-containing octamer-binding protein (NONO) is a multifunctional member of the Drosophila behavior/human splicing (DBHS) protein family with DNA- and RNA-binding activity. NONO is highly expressed in various types of cancer, and excessive *O*-GlcNAcylation has also been implicated in tumorigenesis. Although recent studies revealed that NONO is *O*-GlcNAcylated and that this modification is involved in DNA damage repair, it remains unknown whether *O*-GlcNAcylation of NONO regulates cancer cell proliferation. Additionally, little is known about the effect of *O*-GlcNAcylation on other biological properties of NONO. In this study, we identify Thr440 as the primary NONO *O*-GlcNAcylation site and demonstrates its crucial role in the assembly of paraspeckles, an important subnuclear compartment that facilitates NONO-dependent transcriptional regulation in mammalian cells. Moreover, we found that *O*-GlcNAcylation of NONO is required to maintain the expression of genes related to microtubule cytoskeleton organization involved in mitosis and to suppress the expression of genes related to cellular response to type I interferon. Regarding the regulation of these genes, depletion of NONO *O*-GlcNAcylation at Thr440 significantly inhibited the proliferation of colon cancer cells. Collectively, our findings highlight NONO *O*-GlcNAcylation as a key regulator modulating paraspeckle formation and as a candidate therapeutic target in colon cancer.

## Introduction

The *Drosophila* behavior/human splicing (DBHS) protein family includes splicing factor proline/glutamine-rich (SFPQ), paraspeckle protein component 1 (PSPC1), and non-POU domain-containing octamer-binding protein (NONO), which are versatile nucleoplasmic proteins known for their multifaceted roles in gene regulation. The core region of these proteins shares the highly conserved DBHS domain, which includes two tandem RNA recognition motifs and a NONA/paraspeckle domain that facilitates dimerization or oligomerization of DBHS protein monomers [1]. The DBHS proteins are splicing factors, transcription enhancers/repressors, and DNA repair factors/co-factors that orchestrate various aspects of gene regulation [2]. Along with the *NEAT1_2* long non-coding RNA and more than 40 other proteins, all three DBHS family members localize to a subnuclear mammalian cell compartment known as the paraspeckle [3–5]. The precise function of paraspeckles is still under active investigation, but it is widely accepted that paraspeckles play a pivotal role in gene expression. Paraspeckles retain and sequester nuclear proteins and microRNAs and modulate diverse cellular processes including differentiation, innate immunity, and tumorigenesis [2]. While all three DBHS members are integral core components of paraspeckles, NONO is one of eight proteins that are indispensable for paraspeckle formation [5]. The precise mechanism by which NONO is targeted to the paraspeckle core remains a subject of ongoing investigation.

Reversible post-translational modification of proteins is an important mechanism for regulating gene expression and many other cellular processes. In eukaryotic cells, one such reversible protein modification involves *O*-linked attachment of *N*-acetylglucosamine (GlcNAc) moieties to serine or threonine residues of nuclear, cytoplasmic, and mitochondrial proteins in a process known as *O*-GlcNAcylation which is catalyzed by *O*-GlcNAc transferase (OGT) and removed by *O*-GlcNAcase (OGA). This modification depends on the availability of the donor substrate uridine diphosphate GlcNAc (UDP-GlcNAc), an end-product of the hexosamine biosynthetic pathway (HBP), that in turn depends on metabolic pathways for glucose, amino acids, fatty acids, and nucleotides [6]. Furthermore, because the pathway requires glucose-derived substrates, *O*-GlcNAcylation is highly sensitive to nutrient availability. Recent studies have associated increased protein *O*-GlcNAcylation with tumorigenesis [7–11]. However, the relationships between protein *O*-GlcNAcylation, tumorigenesis, and paraspeckle formation remain unknown.

It has been reported that NONO is dysregulated in various cancer types. For example, NONO has been implicated in breast cancer progression through its ability to promote pre-mRNA splicing of cell proliferation-related genes [12]. NONO also promotes the formation of an oncogenic splice variant of *BIN1*, which enhances hepatocellular carcinogenesis [13]. Although it has been recently reported that NONO is *O*-GlcNAcylated and that this modification plays an important role in DNA damage repair [14], the primary *O*-GlcNAcylation site and the effect of *O*-GlcNAcylation on NONO function in cancer remain elusive.

In this study, we identified threonine 440 (Thr440) as a functionally important *O*-GlcNAcylation site on NONO that is crucial for its interaction with paraspeckle components *NEAT1_2*, SFPQ, and PSPC1. Furthermore, *O*-GlcNAcylation of NONO at Thr440 is required to maintain the expression of genes related to GO categories microtubule cytoskeleton organization involved in mitosis and suppression of cellular response to type I interferon. Notably, colorectal cancer cells expressing NONO T440A, which lacks the Thr440 *O*-GlcNAcylation site, grow less well than cells expressing wild-type NONO. These results suggest that inhibition of *O*-GlcNAcylation of NONO at Thr440 would be a novel therapeutic strategy against the development, growth, and/or progression of colon cancer.

## Results

### The number of NONO-containing paraspeckle-like structures is regulated by cellular *O*-GlcNAcylation status

Previous studies in liver cells show that the abundance of NONO in nuclear speckle-like structures depends on nutrient abundance [15]. Therefore, we investigated the dynamic subnuclear localization of NONO in response to cellular *O*-GlcNAcylation status, which also depends on glucose availability. For this purpose, C57BL/6J mice were subject to 12-hour fasting or 12-hour fasting plus intraperitoneal injection of the OGA inhibitor Thiamet-G, while a non-fasting control group of mice was given *ad libitum* access to regular chow. We harvested liver samples and prepared tissue sections, which were immunostained to visualize the subnuclear distribution of NONO protein. The results show notably fewer NONO-containing paraspeckle-like structures in mice subject to 12-hour fasting than in control non-fasted mice as previously reported (Fig. S1) [15]. Intriguingly, this reduction was not observed when Thiamet-G was administered to 12-h fasted mice (Fig. S1).

**Fig. 1:**
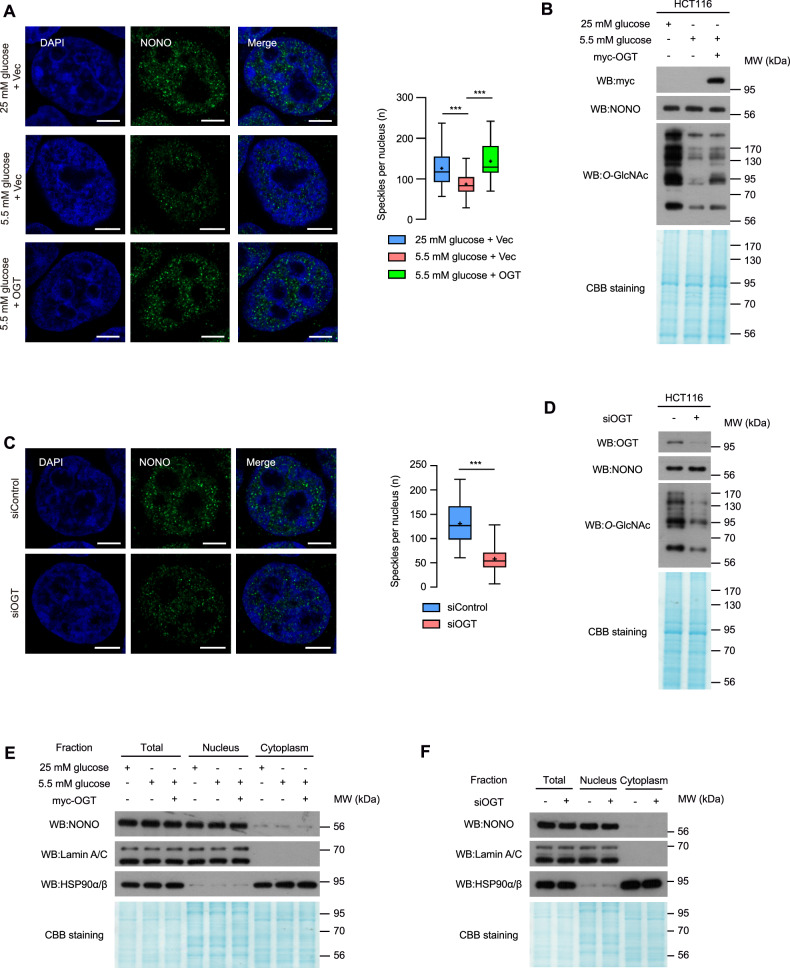
The number of NONO-containing paraspeckle-like structures decreases by lowering cellular *O*-GlcNAcylation. **A** NONO immunostaining on colon cancer cell HCT116 in 25 mM glucose, 5.5 mM glucose, or 5.5 mM glucose transfected with myc-tagged OGT conditions. **B** Cellular *O*-GlcNAcylation status was detected via western blot. **C** NONO immunostaining on colon cancer cell HCT116 in siControl, or siOGT conditions. **D** Cellular *O*-GlcNAcylation status was detected via western blot. **E**, **F** Nuclear and cytoplasmic fractions were analyzed by western blot with relevant antibodies. Lamin A/C and HSP90α/β were used as nuclear and cytoplasmic markers, respectively. Results in (**A**), (**C**) are represented as boxes and whiskers: 10-90 percentile range, “+” sign represents mean, n > 50 nuclei from 3 different biologically independent replicates. ***P < 0.001 (**A**; one-way ANOVA with Tukey’s multiple comparisons test, **C**; Unpaired two-tailed *t*-test). Scale bar represents 5 μm.

To explore whether cellular *O*-GlcNAcylation status influences the incorporation of NONO into paraspeckle-like structures, we used a cell culture model for easier manipulation of nutrient and *O*-GlcNAcylation levels. As a previous study reported that NONO interacts with OGT in HCT116 human colorectal carcinoma cells [8], further experiments were conducted using this cell line. HCT116 cells were cultured in DMEM media containing high glucose (25 mM), low glucose (5.5 mM), or low glucose with overexpression of OGT. Consistent with results obtained in the liver tissues of C57BL/6J mice, the number of NONO-containing paraspeckle-like structures was lower in cells grown under low glucose conditions (Fig. 1A). Remarkably, this downregulation was mitigated in HCT116 cells overexpressing OGT (Fig. 1A). This inhibition of paraspeckle-like structure formation was confirmed by siRNA knockdown of OGT in HCT116 cells (Fig. 1C), human breast adenocarcinoma MCF7 cells, and human non-small cell lung cancer A549 cells (Fig. S2A, C). Importantly, we also demonstrated that the total amount of NONO protein in these cells were similar under all conditions tested (Fig. 1B, D, Fig. S1B, and Fig. S2B, D, E–H). In addition, nuclear/cytosolic fractionation reveals that subcellular localization of NONO was not affected by *O*-GlcNAc modulation (Fig. 1E, F). Together, these results suggest that the formation of NONO-containing paraspeckle-like structure depends specifically on the status of cellular *O*-GlcNAcylation.

### NONO is *O*-GlcNAcylated at Thr440

**Fig. 2:**
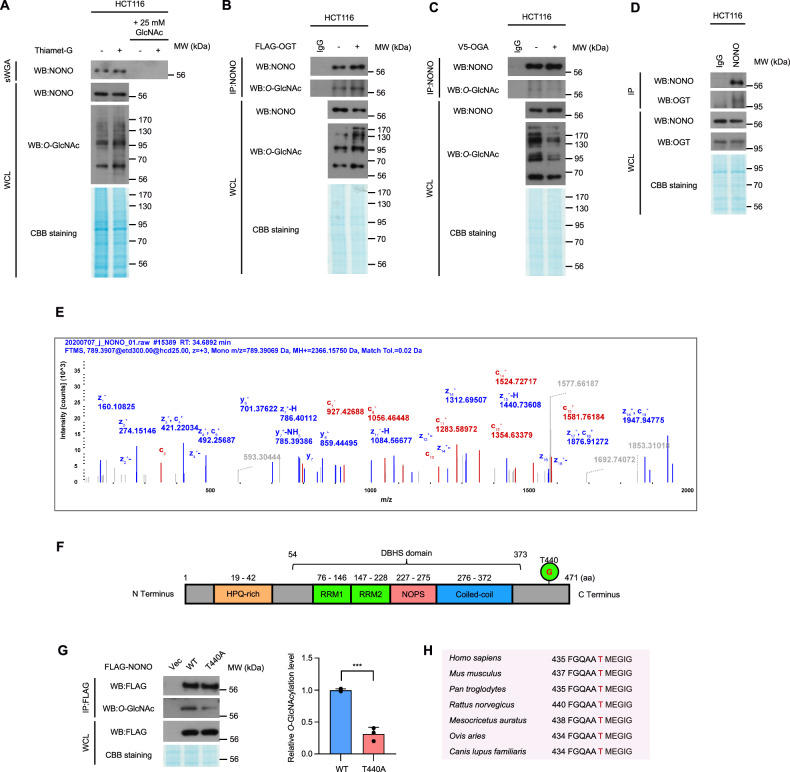
NONO *O*-GlcNAcylation site was identified as Thr440. **A** sWGA precipitation was performed to evaluate immunoprecipitated NONO level upon with or without GlcNAc competition. Immunoprecipitated NONO protein level was tested by western blot analysis. **B** Validation of *O-*GlcNAcylation with endogenously-expressed NONO in HCT116 cells. The NONO *O-*GlcNAcylation in HCT116 was higher in cells transfected with OGT. **C** The NONO *O-*GlcNAcylation in HCT116 was lower in cells transfected with OGA. **D** A co-IP assay was performed to evaluate the interactions between NONO with OGT. HCT116 WCLs were subjected to IP with normal rabbit IgG of anti-NONO antibody. **E** Mass spectrometry (MS) analysis identifies the *O*-GlcNAcylation site in NONO. EThcD spectra of *O-*Glycopeptide FGQAATMEGIGAIGGTPPAFNR from human NONO is shown. The site of *O*-GlcNAc modification was identified as threonine 440. The c and z fragments detected are as indicated in the sequence. **F** Human NONO protein structure. *O*-GlcNAcylation was indicated at threonine 440. (RRM; RNA recognition motif, NOPS; NONA/Paraspeckle). **G** IP assay to compare the *O*-GlcNAcylation levels of wild-type NONO with T440 to A mutant. 10 ug of either FLAG-tagged NONO WT or mutant were transfected into HEK293 cells and lysed after 24 h. NONO *O*-GlcNAcylation level was normalized to the immunoprecipitated NONO protein level (*n* = 3 per condition). **H** Sequence information indicates that NONO threonine 440 is a highly conserved sequence in mammals. Data are presented as mean ± SD; ***P < 0.001, Unpaired two-tailed *t*-test.

Previous studies have demonstrated that NONO is *O*-GlcNAcylated [14] and our findings, as presented above, show that the incorporation of NONO into paraspeckle-like structures is influenced by cellular *O*-GlcNAcylation status. These results suggest that *O*-GlcNAcylation of NONO may directly influence the formation of NONO-containing paraspeckle-like structures. Therefore, we sought to identify the major *O*-GlcNAcylation site on NONO protein. Initially, we confirmed NONO *O*-GlcNAcylation in HCT116 cells. For this purpose, NONO was affinity purified using succinylated wheat germ agglutinin (sWGA) from extracts of HCT116 cells grown in the presence or absence of Thiamet-G. The results of this experiment confirm that endogenous NONO is *O*-GlcNAcylated and that treatment with Thiamet-G substantially increased NONO *O*-GlcNAcylation in HCT116 cells (Fig. 2A). The specificity of our purification technique was validated by the complete abolition of purified NONO when it was competed with 25 mM GlcNAc (Fig. 2A). In addition, endogenous NONO was immunoprecipitated with a NONO-specific antibody from cells overexpressing OGT or OGA, and its *O*-GlcNAcylation status was analyzed with an *O*-GlcNAc-specific antibody. The results show that *O*-GlcNAcylation of NONO was higher or lower, respectively, in cells overexpressing OGT or OGA (Fig. 2B, C). The immunoprecipitation results also demonstrated that NONO interacts with OGT (Fig. 2D).

To identify amino acid residues that are *O*-GlcNAcylated in NONO using mass spectroscopy (MS), we transiently overexpressed FLAG-tagged NONO in HEK293 cells and confirmed NONO *O*-GlcNAcylation (Fig. S3A, B). MS analysis detected *O*-GlcNAcylation of NONO only at Thr440 in the C-terminal disordered region of the protein (Fig. 2E and Dataset S1), as illustrated Fig. 2F. To assess that Thr440 is the primary *O*-GlcNAcylation site in NONO, point mutant NONO T440A was generated, in which Thr440 is replaced with alanine. FLAG-NONO wild type (WT) and FLAG-NONO T440A were overexpressed in HEK293 cells, followed by immunoprecipitation with a FLAG-specific antibody. *O*-GlcNAcylation of FLAG-NONO was then analyzed with an *O*-GlcNAc-specific antibody. The results revealed that FLAG-NONO T440A showed a significant reduction in *O*-GlcNAcylation compared to FLAG-NONO WT (Fig. 2G), suggesting that this site is the primary *O*-GlcNAc site of NONO. Given that Thr440 is highly conserved among mammalian NONO homologs, we conclude that *O*-GlcNAcylation of Thr440 (or its equivalent) in NONO could be a widely conserved mechanism for regulating NONO in vertebrate species (Fig. 2H).

### Thr440 *O*-GlcNAcylation of NONO is responsible for paraspeckle complex formation

**Fig. 3:**
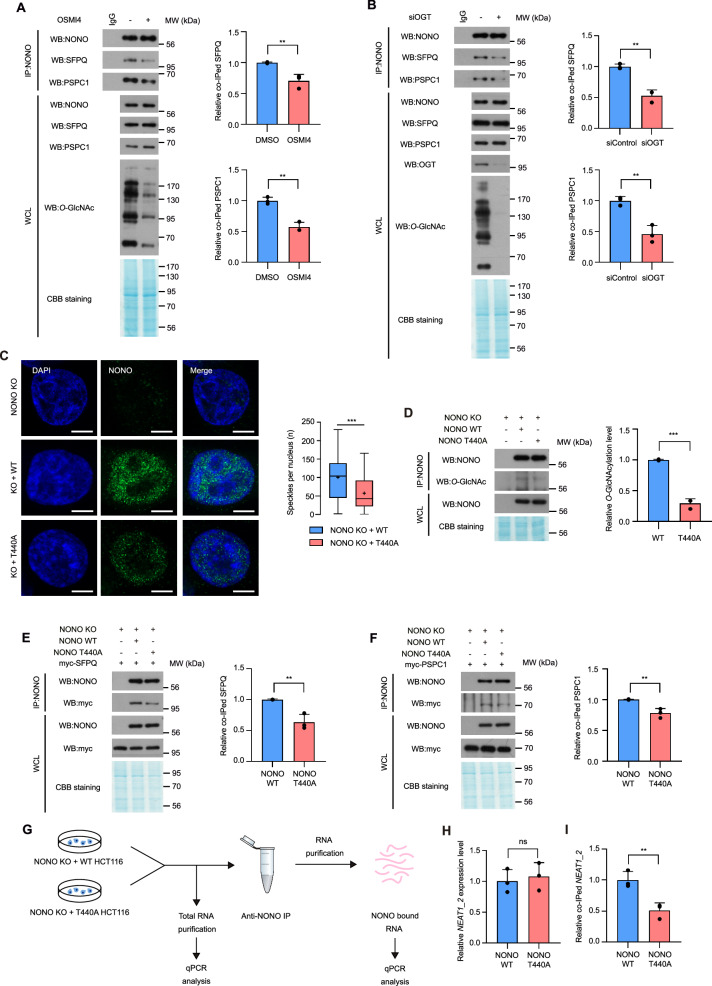
Thr440 *O*-GlcNAcylation of NONO is necessary for paraspeckle components interaction. **A**, **B** A co-IP assay was performed to evaluate the interactions between NONO with SFPQ, and PSPC1. HCT116 cells were treated with OSMI4 or siOGT and lysed after 24 h or 48 h, respectively. WCLs were subjected to IP with anti-NONO antibody. Relative co-immunoprecipitated SFPQ, PSPC1 level was normalized to NONO (*n* = 3 per condition). **C** NONO immunostaining on NONO knockout, stably recovered with NONO WT, or T440A colon cancer cell HCT116. *n* > 50 nuclei from 3 different biologically independent replicates. **D** *O*-GlcNAcylation levels of stably expressed WT NONO with T440A mutant were compared (*n* = 3 per condition). **E**, **F** A co-IP assay was performed to evaluate the interactions between NONO WT or T440A with SFPQ, and PSPC1. NONO KO HCT116 cells were transfected with FLAG-tagged NONO WT or T440A and transfected with myc-tagged SFPQ or PSPC1 and lysed after 24 h. Immunoprecipitation was performed with FLAG^®^-agarose. Relative co-immunoprecipitated SFPQ, PSPC1 level was normalized to NONO WT or T440A (*n* = 3 per condition). **G** RIP analysis was performed to evaluate co-immunoprecipitated lncRNA *NEAT1_2* level with NONO WT or T440A. Schematic of RNA immunoprecipitation experimental procedure. **H** Total *NEAT1_2* level was evaluated by qPCR analysis (*n* = 3 per condition). **I** Co-immunoprecipitated *NEAT1_2* level with NONO WT or T440A protein was evaluated by qPCR analysis (*n* = 3 per condition). Data are presented as mean ± SD; **P < 0.01, ***P < 0.001, Unpaired two-tailed *t*-test. Scale bar represents 5 μm.

Given the influence of *O*-GlcNAcylation on the formation of NONO-containing paraspeckle-like structures, we proceeded to investigate whether *O*-GlcNAcylation could impact paraspeckle formation, where DBHS proteins primarily reside. To explore this, we performed co-immunoprecipitation experiments involving NONO, SFPQ, and PSPC1 in extracts from HCT116 cells under conditions of altered cellular *O*-GlcNAcylation levels. The findings revealed that significantly less SFPQ and PSPC1 co-immunoprecipitated with NONO from cells treated with OSMI4, a specific inhibitor of OGT (Fig. 3A). Similar results were obtained when OGT was knocked down with OGT-targeted siRNA (Fig. 3B) and when the co-immunoprecipitation experiments were performed in MCF7 (Fig. S4A, B) or A549 cells (Fig. S4C, D), demonstrating that cellular *O*-GlcNAcylation status influences interactions between DBHS proteins in various types of cancer cells. Next, to investigate whether *O*-GlcNAcylation of NONO at Thr440 affects interactions between DBHS proteins, we generated NONO knockout HCT116 cells using CRISPR-Cas9 mutagenesis. We then stably expressed either NONO WT or T440A in these cells and confirmed stably expressing NONO WT contained more NONO-containing paraspeckles than cells expressing NONO T440A or NONO knockout HCT116 cells (Fig. 3C). NONO *O*-GlcNAcylation level was also higher in stably expressed WT compared to T440A NONO (Fig. 3D). Next, we performed the co-immunoprecipitation experiments in these cells. The results demonstrated that less SFPQ and PSPC1 co-immunoprecipitated with NONO T440A than with NONO WT (Fig. 3E, F). Because Thr440 is in the C-terminal disordered region of NONO (Fig. 2F), while the DBHS domain is responsible for DBHS protein dimerization [2], we questioned how the *O*-GlcNAcylation of NONO at Thr440 could promote interactions between DBHS proteins. To investigate this, we performed RNA immunoprecipitation followed by quantitative PCR (RIP-qPCR) to assess the interaction between NONO and *NEAT1_2* long non-coding RNA, which plays a crucial role as the structural scaffold of paraspeckles (Fig. 3G). The total *NEAT1_2* expression level was unchanged in cells expressing NONO WT or T440A (Fig. 3H). However, significantly less *NEAT1_2* RNA were co-immunoprecipitated with NONO T440A than with NONO WT (Fig. 3I). These results suggest that *O*-GlcNAcylation of NONO at Thr440 is likely required for the formation of the paraspeckle complex.

### *O*-GlcNAcylation of NONO at Thr440 is required for maintaining the expression of genes related to microtubule cytoskeleton organization and for suppressing the expression of genes related to cellular response to type I interferon

**Fig. 4:**
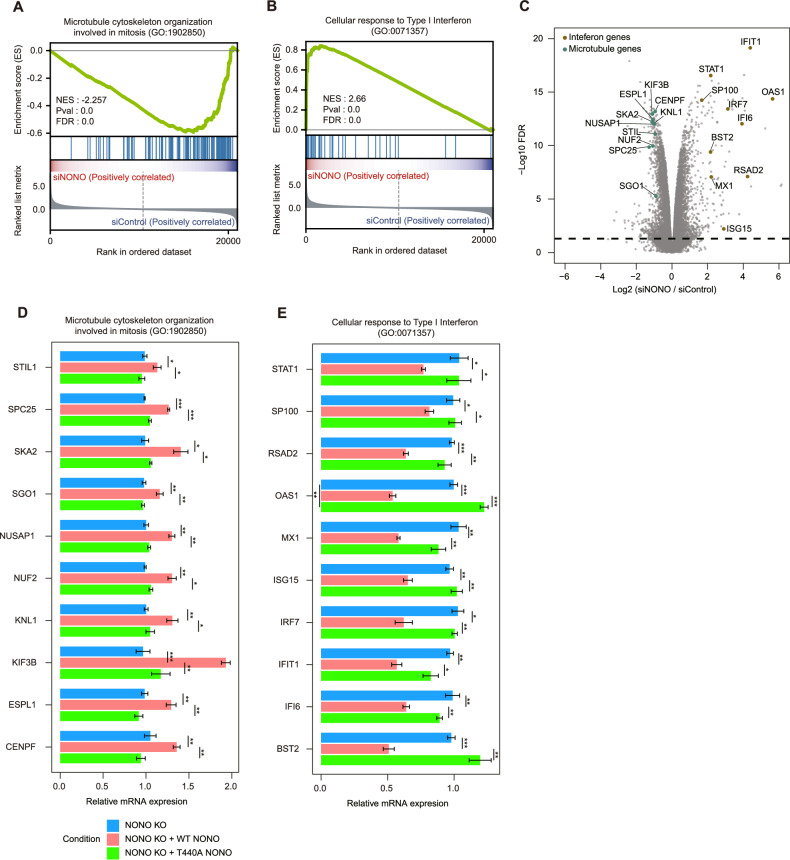
Modifying NONO *O*-GlcNAcylation status results in transcriptional profile alteration. **A**, **B** GSEA(Gene Set Enrichment Analysis) plots of biological functions associated with gene groups whose expression changes in NONO knockdown by siRNA. **C** Volcano plot for differentially expressed gene (DEG) analysis. Genes related to microtubule cytoskeleton organization were downregulated in NONO knock-downed HCT116. Conversely, genes related to cellular response to type I interferon were upregulated in same condition. **D**, **E** qPCR was performed to evaluate the genes associated with microtubule cytoskeleton organization involved in mitosis or cellular response to Type I interferon (*n* = 3 per condition). Data are presented as mean ± SD; *P < 0.05, **P < 0.01, ***P < 0.001 (**(D**, **E)**; one-way ANOVA with Tukey’s multiple comparisons test).

Considering the role of NONO-containing paraspeckles as a transcriptional regulator, we hypothesized that knockdown of NONO might cause detectable changes in the global transcriptome. This idea was tested by comparing expression of putative NONO target genes in HCT116 cells treated with control siRNA (siControl) or NONO-targeted siRNA (siNONO). The results revealed dynamic regulation of gene expression and identified differential expression of genes related to specific GO terms in siNONO-treated cells (Dataset S2). The analysis focused on genes related to two GO terms, the first being “microtubule cytoskeleton organization involved in mitosis” (GO_1902850). The results showed that genes related to this GO term were differentially downregulated in siNONO-treated cells (Fig. 4A). One interpretation of this result is that NONO-stimulated transcription of these genes supports the high rate of cell division in cancer cells by maintaining microtubule organization during mitosis, a possibility that is consistent with the proposed role of NONO in cancer [16, 17]. The second GO term of interest was “cellular response to type I interferon” (GO_0071357). Genes related to this GO term were differentially upregulated in siNONO-treated samples (Fig. 4B). Type I interferons are associated with tumor inhibition and immune system activation, making them a focus of clinical anti-cancer strategies [18]. Thus, genes related to both of these GO terms are linked to cancer. In related experiments, the expression of select genes was analyzed by qPCR in NONO knockout HCT116 cells stably expressing NONO WT or NONO T440A. qPCR analysis was performed for the top 10 genes related to each of the two GO terms mentioned above, excluding genes that belong to similar functional classes based on differentially expressed gene (DEG) analysis (Fig. 4C and Dataset S3). Surprisingly, expression of genes related to “microtubule cytoskeleton organization involved in mitosis” was significantly higher in NONO knockout cells stably expressing NONO WT than in NONO knockout cells stably expressing NONO T440A or control NONO knockout cells (Fig. 4D). In contrast, expression of genes associated with the “cellular response to type I interferon” was lower in NONO knockout cells stably expressing NONO WT than in NONO knockout cells stably expressing NONO T440A or control NONO knockout cells (Fig. 4E). Consistent with these results, knocking down of SFPQ (Fig S5B), another indispensable protein for paraspeckle formation [5], also led to the abolishment of NONO-containing paraspeckle (Fig. S5A) and the downregulation of microtubule organization-related genes (Fig. S5C). Next, we investigated whether paraspeckle formation or altered gene expression could be rescued independently of NONO or its *O*-GlcNAcylation status. To test this, we treated HCT116 cells with siRNA targeting OGT and overexpressed WT NONO (Fig. S5H). We then immunostained PSPC1, the first identified marker protein for paraspeckles [5], to observe subnuclear speckles avoiding bias caused by NONO overexpressing. Although the number of PSPC1-containing subnuclear speckles was reduced after OGT knockdown, overexpression of NONO in *O*-GlcNAc-challenged HCT116 cells could not rescue the formation of these speckle-like structures (Fig. S5G). qRT-PCR analysis revealed that OGT knockdown also suppressed microtubule-related gene expression, which could not be restored by WT NONO overexpression (Fig. S5I). Furthermore, overexpression of SFPQ in HCT116 stably expressing T440A NONO (Fig. S5E) failed to promote the formation of NONO-containing speckles (Fig. S5D) or enhance the expression of microtubule-related genes (Fig. S5F). These findings suggest that NONO *O*-GlcNAcylation at Thr440 has a potentially significant impact on tumorigenesis by fine-tuning gene transcription, through the formation of NONO-containing paraspeckles.

### *O*-GlcNAcylation of NONO at Thr440 promotes cancer cell proliferation

**Fig. 5:**
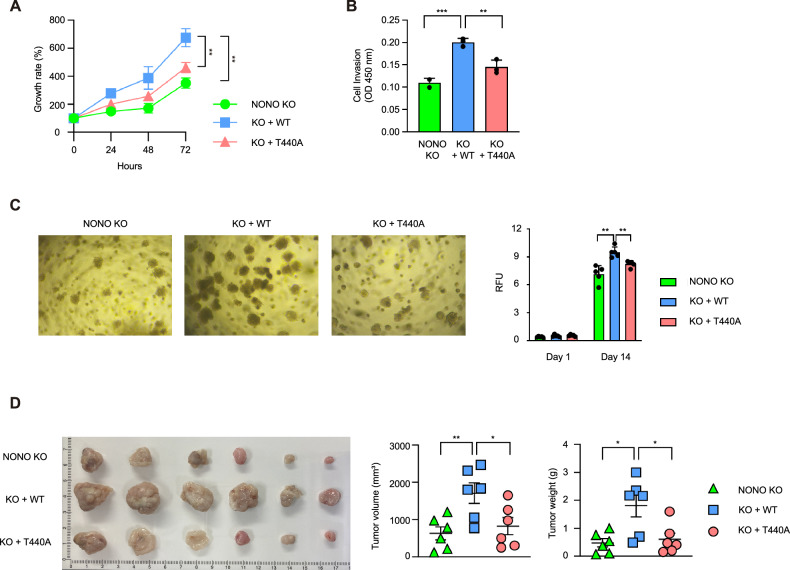
Thr440 *O*-GlcNAcylation of NONO promotes cancer cell invasion, colony formation, and proliferation. **A** WST-8 assay was performed to compare cell proliferation between NONO knockout, NONO WT-expressing, or NONO T440A-expressing HCT116 cells (*n* = 3 per condition). **B** Transwell invasion assay was performed and invaded cells were measured by WST-8 assay (*n* = 3 per condition). **C** NONO knockout, NONO WT-expressing, or NONO T440A-expressing HCT116 cells were cultured for soft agar colony formation assay. Colony formation was examined under a light microscope. Colony formation was measured by using a 485/520 nm filter set (*n* = 5 per condition). **D** BALB/c nude mice (*n* = 6 per group) were injected with 1 × 10^7^ of the indicated cells subcutaneously. Tumor volume and weight were measured 60 days after injection. Data are presented as mean ± SD; *P < 0.05, **P < 0.01, ***P < 0.001, one-way ANOVA with Tukey’s multiple comparisons test.

**Fig. 6:**
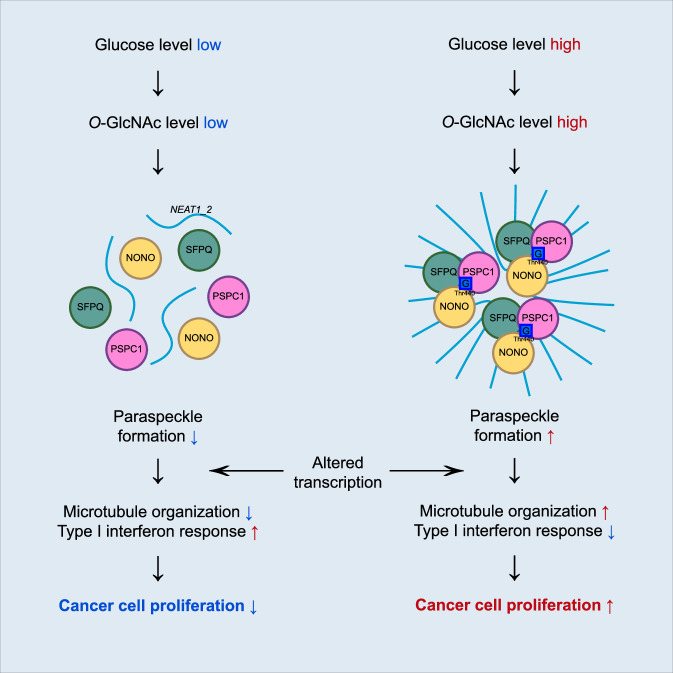
Schematic of the proposed model depicting *O*-GlcNAcylation of NONO at Thr440 and its role in cancer cell proliferation. In cancer cells, a relatively high level of glucose allows more cellular *O*-GlcNAc modification. *O*-GlcNAcylation of NONO at Thr440 enhances the paraspeckle formation. Upregulated paraspeckle formation promotes transcriptional alteration, leading to cancer cell proliferation.

To explore NONO’s potential role in cancer in greater detail, the rate of cell proliferation was measured in HCT116, MCF7, and A549 cells with or without exposure to siNONO to deplete endogenous NONO. The results showed that depletion of NONO protein significantly delayed proliferation in all three cell types (Fig. S6A–C). Next, we delved into whether Thr440 *O*-GlcNAcylation of NONO is involved in cancer cell proliferation. We employed previously established NONO knockout HCT116 cells, which were stably reconstituted with either NONO WT or the T440A mutant. These cells were subjected to a cell proliferation assay. Significantly, cells expressing NONO WT proliferated markedly faster than cells expressing NONO T440A or the control NONO knockout HCT116 cells (Fig. 5A). Consistent with the fact that most of the top 10 NONO-responsive genes related to “microtubule cytoskeleton organization involved in mitosis” promote invasion of cancer cells [19–26], cell invasion assays showed that NONO WT but not NONO T440A increased the invasiveness of NONO knockout HCT116 cells (Fig. 5B). Additionally, we conducted soft agar colony formation assays to observe the growth of cell clusters. Similar results were obtained with an assay for colony formation in soft agar, with larger colonies forming with NONO knockout HCT116 cells expressing NONO WT (Fig. 5C). The ability of NONO to stimulate tumor proliferation in vivo was also examined using a male BALB/c nude mouse xenograft model. Consistent with the data presented above, cells expressing NONO WT formed significantly larger xenograft tumors (by volume and weight) than NONO knockout or T440A-expressing HCT116 cells (Fig. 5D). In summary, this study suggests that *O*-GlcNAcylation of NONO at Thr440 contributes to paraspeckle formation, resulting in dynamic transcriptional modulation and ultimately promoting cancer cell proliferation (Fig. 6).

## Discussion

High levels of *O*-GlcNAcylation of serine and/or threonine residues in nuclear, cytoplasmic, and mitochondrial proteins are correlated with poor prognosis for several types of cancers. NONO, a member of the DBHS protein family, is known to be involved in diverse nuclear processes such as pre-mRNA splicing, non-homologous end joining (NHEJ), and transcriptional activation or repression. Recently, the role of NONO in tumorigenesis has been highlighted. NONO is also known as an *O*-GlcNAc-modified protein, which is responsible for regulating chromatin association and dissociation of NONO during the NHEJ pathway [14]. However, how this modification of NONO contributes to tumorigenesis remains to be elucidated. This study identifies Thr440 as the major *O*-GlcNAcylation site of NONO and provides insight into the functional significance of this modification, including its impact on paraspeckle formation, gene expression, colon cancer cell proliferation, and tumorigenesis.

Paraspeckles are subnuclear bodies harbored in the interchromatin region of mammalian cell nuclei. They are known to modulate gene expression in diverse contexts including development, the innate immune response, and stress. Several groups have demonstrated that paraspeckles promote tumor progression and chemoresistance, by repressing apoptosis in cancer cells [27], preventing hypoxic response [28], and moderating replication stress [29]. Moreover, there are evidences indicating the relationship between *NEAT1*, the skeletal lncRNA of paraspeckle, and microtubule stabilization [30] or cancer proliferation [31]. Consistent with these results, this study identified an association between paraspeckle assembly, tumorigenesis, and transcriptional regulation. The results show a reduced number of paraspeckle-like structures containing NONO in cells from fasted mice (Fig. S1A), whereas a similar reduction in cells grown in culture with low concentrations of glucose was rescued by overexpression of OGT (Fig. 1A), strongly supports the link between *O*-GlcNAcylation and paraspeckle formation. Importantly, the identification of Thr440 as the primary *O*-GlcNAcylation site on NONO provides insight into the molecular basis of this regulatory mechanism.

Although *O*-GlcNAcylation of NONO is strongly decreased in the NONO T440A mutant, the persistence of the modification (Fig. 2G) suggests that additional *O*-GlcNAcylation sites on NONO exist but were not detected by MS analysis in our study. Because technical challenges hinder the identification of these sites by MS analysis, alternative techniques and additional studies will be needed for this purpose. In addition, our study was not able to explain the direct mechanism how Thr440 *O*-GlcNAcylation of NONO regulates the formation of NONO-containing paraspeckles, resulting in tumor growth. Furthermore, as DBHS proteins are structurally similar, it seems possible that SFPQ and PSPC1 also be regulated by *O*-GlcNAcylation (Fig. S7). Future studies adopting other methods such as RNA fluorescence in situ hybridization (RNA-FISH), electron microscopy in situ hybridization (EM-ISH) or structural analysis would be needed to address these concerns, the broader implications of *O*-GlcNAcylation of DBHS proteins in different cancer types, and to explore the potential of DBHS *O*-GlcNAcylation as a therapeutic target in cancer. Significantly, the sensitivity of paraspeckle formation to nutrient availability links paraspeckles to cellular metabolism, and *O*-GlcNAcylation is linked to the dynamic organization of nuclear structures.

In this study, the downstream effects of NONO *O*-GlcNAcylation on gene expression were analyzed using RNA-sequencing and qPCR. The results revealed a link between NONO *O*-GlcNAcylation at Thr440 and the expression of genes related to two GO terms: microtubule cytoskeleton organization involved in mitosis and cellular response to type I interferon (Fig. 4A–E). These findings suggest a connection between the *O*-GlcNAcylation status of NONO, cell cycle progression, and immune response, which is consistent with the results of functional assays. For example, NONO *O*-GlcNAcylation at Thr440 is linked to colon cancer cell proliferation, invasion, and colony formation in vitro, as well as tumor proliferation in an in vivo xenograft tumor model (Fig. 5A–D). We highlight the importance of NONO *O*-GlcNAcylation in promoting colon cancer cell proliferation and aggressiveness because our results provide compelling evidence that Thr440 *O*-GlcNAcylation of NONO has the potential as a novel therapeutic target for inhibiting colon tumor proliferation.

At a broader level, the present study provides insight into the intricate regulatory networks governing gene expression and cellular processes in cancer cells. The interplay between *O*-GlcNAcylation, paraspeckle formation, and selective up- or downregulation of gene expression, as outlined in this study, demonstrates that DBHS proteins, especially NONO, play complex roles in cancer biology. We are hopeful that this study will lead to novel avenues in cancer research, potentially involving *O-*GlcNAcylation of NONO as a cancer therapeutic target.

## Methods

### Laboratory animals

Male C57BL/6J and BALB/c nude mice were purchased from DBL (DBL Co., Ltd., Eumseong, South Korea). The mice were housed in a controlled environment with unrestricted access to water and standard rodent chow, under conditions of 23 ± 3 °C temperature, 55 ± 10% humidity, and a 12-h light/dark cycle. The Institutional Animal Care and Use Committees of the Laboratory Animal Research Center at Yonsei University approved the experiments (IACUC-A-202107-1286-02, IACUC-A-202205-1470-01).

### Cell cultures

HEK293, HCT116, MCF7 cells were cultured in Dulbecco’s Modified Eagle’s Medium (Welgene, #LM 001-05, South Korea) supplemented with 10% fetal bovine serum (Gibco, #16000-044, USA), 100 U/mL penicillin and 100 μg/mL streptomycin (Gibco, #15140122, USA). A549 cells were cultured in RPMI-1640 Medium (Hyclone, #SH30255.01, USA). All cell lines were incubated in 5% CO_2_ at 37 °C. All cells were tested for mycoplasma contamination using a mycoplasma detection polymerase chain reaction (PCR) test (Bionics, South Korea).

### Transfection and plasmids

Wild-type NONO-expressing plasmid was prepared by PCR and subcloned into the EcoRI and BamHI sites of the p3XFlag-CMV7.1 expression vector (Sigma-Aldrich, USA). pCMV7.1-3XFlag-NONO T440A (Thr 440 mutated to Ala) construct was generated by using Muta-Direct Site-Directed Mutagenesis Kit (iNtRON, #15071, South Korea). The mutations were confirmed by DNA sequence analyses (Bionics, South Korea). Human SFPQ and PSPC1 cDNA were kindly provided by Prof. Jong-Bok Yoon (Yonsei University, Seoul, South Korea) and subcloned into the pcDNA3.1-mycHis(-) A expressing vector (Invitrogen, USA), respectively. pMSCV-hygro/FLAG vector was kindly provided by Prof. Jaewhan Song (Yonsei University, Seoul, South Korea). DNA transfection was performed by PEI based on the manufacturer’s protocol.

To knockdown OGT or NONO via siRNA interference, cells were transfected with siRNAs targeting OGT or NONO with Lipofectamine RNAiMAX (Invitrogen, USA) according to the manufacturer’s transfection protocol. The siRNA sequences were: siControl (duplex, Cat. SN-1002, Bioneer, Korea), siOGT (sense, 5′-UAAUCAUUUCAAUAACUGCUUCUGC(dTdT)-3′, antisense, 5′-GCAGAAGCAGUUAUUGAAAUGAUUA(dTdT)-3′), siNONO (sense, 5′-GGAAGCCAGCUGCUCGGAAAGCUCU-3’, antisense, 5′-AGAGCUUUCCGAGCAGCUGGCUUCC-3′).

### Stable cell line establishment

NONO knockout was achieved in HCT116 cells through the CRISPR-Cas9 system, following previously documented methods [32, 33]. In brief, a synthesized sgRNA targeting NONO (sequence: GACCAGTTAGATGATGAAGA) was inserted into the pSpCas9(BB)-2A-Puro (PX459) vector (Addgene, #62988, USA). The sgRNA/Cas9 plasmid was transfected into HCT116 cells using lipofectamine 2000 (Invitrogen, #11668027, USA), and subsequent selection of cells was carried out using puromycin. For the introduction of wild-type and T440A mutant NONO DNAs, they were cloned into the pMSCV-hygro/FLAG vector. The cloned retroviral DNA constructs were transfected with packaging plasmids pCMV-VSV-G and pCMV-Gag-Pol into HEK293T cells employing lipofectamine 2000. After 48 h, retroviral supernatants were harvested, filtered with a 0.45 μm filter, and then utilized to infect the previously generated NONO knockout HCT116 cells supplied with 4 μg/mL of polybrene. The virally infected cells were selected with hygromycin.

### Immunostaining

For liver immunostaining, samples were obtained from the livers of C57BL/6J mice belonging to three randomly divided groups: control, 12-hour fasting, and combined 12-hour fasting with an intraperitoneal injection of 40 mg/kg Thiamet-G. The primary liver lobes were roughly cut into pieces and embedded in Tissue-Tek O.C.T compound (Sakura, #4583, USA). The embedded liver tissue was then sectioned into 5 μm thickness and placed on 26*76*1.0 mm microscope slides (Marienfeld Superior, Germany). Subsequently, the liver sections were air-dried overnight at room temperature and stored at −80 °C for extended preservation. For the immunostaining procedure, frozen sections underwent washing with PBST to eliminate any remaining O.C.T. They were then fixed with chilled methanol for 10 min at −20 °C. Sections were then washed 3 times with PBS for 5 min each. After the wash, the sections were subjected to overnight incubation with an anti-NONO rabbit polyclonal antibody (Abcam, #ab70335, USA) at a 1:200 dilution in PBS. After 3-time washes in PBS, the liver sections were exposed to the secondary antibody Alexa Fluor 488 conjugate anti-rabbit IgG (Invitrogen, #A-11008, USA) at a 1:500 dilution in PBS for 1 h.

Cells were grown on 18*18 mm cover glasses (Marienfeld Superior, Germany) for immunostaining. They were washed with PBS and fixed with 4% paraformaldehyde for 10 min at RT. After washing with PBS again, the cells were permeabilized with 0.5% Triton-X-100 in PBS for 10 min, followed by 2 times washing with 0.1% Triton-X-100 in PBS. Then they were blocked with 3% BSA in PBS for 30 mins at RT. After blocking, the cells were subjected to 2 h incubation at 37 °C with anti-NONO rabbit polyclonal antibody (Abcam, #ab70335, USA) or anti-PSPC1 rabbit polyclonal antibody (Abcam, #ab104238, USA) at a 1:200 dilution in PBS containing 3% BSA. They were 3-time washed with 0.1% Triton-X-100 in PBS, followed by 1 h incubation with the secondary antibody Alexa Fluor 488 conjugate anti-rabbit IgG (Invitrogen, #A-11008, USA) at a 1:500 dilution in 3% BSA in PBS, and washed 3 times again with 0.1% Triton-X-100 in PBS. The liver sections or the cells were mounted with Vectashield HardSet Antifade Mounting Medium with DAPI (Vector Laboratories, #H-1500, USA). All images were collected on a Zeiss LSM 980 confocal microscope (Carl Zeiss) and analyzed with Icy software [34] using spot detector plugin.

### Western blotting and immunoprecipitation

For FLAG immunoprecipitation (IP), cellular lysates underwent a 2 h incubation with agarose-conjugated anti-FLAG antibody (MBL, Woburn, USA) at room temperature (RT). In the case of NONO IP, the anti-NONO antibody (Abcam, #ab70335, USA) was incubated overnight at 4 °C, followed by incubation with agarose-conjugated protein A/G (Santa Cruz) overnight at 4 °C. As a control for immunoprecipitation (IP), normal rabbit IgG (Cell Signaling Technology, #2729, USA) antibody was used. The purified proteins in the IP precipitates underwent three washes with the wash buffer (150 mM NaCl, 2 mM EGTA, 2 mM MgCl2, 20 mM HEPES, pH 7.4, and 0.1% NP-40) and were eluted with 2× sodium dodecyl sulfate (SDS) loading buffer at 95 °C for 5 min. The eluates were subsequently analyzed via western blot using specific antibodies. For RNA-IP, Trizol was added directly to the beads after immunoprecipitation and subjected to RNA extraction following the manufacturer’s protocol. sWGA precipitation was performed as previously described [35]. Briefly, 2 mg of total cell lysates underwent an overnight incubation with agarose-conjugated sWGA (Vector Laboratories, Burlingame, CA, USA) at 4 °C, with or without 25 mM GlcNAc (Sigma, #A8625, USA).

Cell lysis was performed using RIPA buffer (50 mM Tris-HCl, pH 7.4, 150 mM NaCl, 2 mM EDTA, 1% NP-40, 0.1% SDS, and 0.5% sodium deoxycholate) or 1% NP-40 lysis buffer (50 mM Tris-HCl, pH 7.4, 150 mM NaCl, 2 mM EDTA, and 1% NP-40) for western blotting, or Pierce IP lysis buffer (25 mM Tris-HCl, pH 7.4, 150 mM NaCl, 1 mM EDTA, 1% NP-40, and 5% glycerol) for immunoprecipitation, supplemented with protease inhibitor cocktail (Roche, Mannheim, Germany). A total of 10 μg of cellular lysate was loaded onto SDS-polyacrylamide electrophoresis gels. Following antibody incubation, signal detection utilized WestGlow chemiluminescent substrate (Biomax, #BWE0200, Korea) and FUJI RX-N X-ray film (FUJI, Japan). Signal quantification was performed using ImageJ software.

The antibodies used for western blotting or IP included anti-FLAG (MBL, #PM020), anti-HSP90α/β (Santa Cruz, #sc-7947), anti-Lamin A/C (Cell Signaling, #2032S), anti-c-Myc (Santa Cruz, #sc-40), anti-NONO (Abcam, #ab70335 for IP), anti-NONO (Santa Cruz, #sc-136296 for WB), anti-SFPQ (Santa Cruz, #sc-374502), anti-PSPC1 (Santa Cruz, #374181), anti-*O*-GlcNAc (Thermo Fisher Scientific, #MA1-072), and anti-OGT (Sigma, #O6264). Secondary antibodies included goat anti-rabbit IgG (#111-035-003, Jackson Laboratories, Bar Harbor, ME, USA), mouse anti-rabbit IgG (light-chain specific, #211-032-171, Jackson Laboratories), goat anti-mouse IgG (#115-035-003, Jackson Laboratories), and goat anti-mouse IgG (light chain specific, #115-035-174, Jackson Laboratories).

### Nuclear and cytosol fractionation

Cells were incubated in ice-cold cytosolic lysis buffer (10 mM HEPES pH 7.9, 10 mM KCl, 0.1 mM EDTA, 0.1 mM EGTA, and 1 mM DTT) supplemented with protease inhibitor cocktail for 15 min, vortex after adding 0.5% NP-40 for 10 s and centrifuged at 4,000 rpm for 4 min at 4 °C. The supernatant containing the cytoplasmic extract was collected to new ep tube. The pellet was washed with cytosolic lysis buffer without DTT and centrifuged at 4000 rpm for 3 min at 4 °C, then incubated in nuclear lysis buffer (20 mM HEPES pH 7.9, 0.4 M NaCl, 1 mM EDTA, 1 mM EGTA, and 1 mM DTT) supplemented with protease inhibitor cocktail on ice for 30 min with vortexing occasionally and centrifuged at 14,000 rpm for 20 min at 4 °C. The supernatant was collected into fresh ep tube.

### Protein isolation and digestion

HEK293 cells were transfected with NONO tagged with a FLAG, and co-transfected with OGT for hyper-O-GlcNAcylation induction. To further enrich NONO, we employed immunoprecipitation using an anti-FLAG antibody, followed by SDS-PAGE separation and subsequent staining with Coomassie Brilliant Blue reagent. The specific fraction containing NONO was excised from the SDS-PAGE gel and subjected to in-gel tryptic digestion. The targeted protein fraction was then destained using a solution of 50% (v/v) acetonitrile in 25 mM NH_4_HCO_3_. Subsequently, the fraction underwent reduction with 20 mM DTT at 60 °C for 1 h, followed by alkylation with 55 mM iodoacetamide at room temperature for 45 min in the absence of light. The fraction was then digested with trypsin at 37 °C overnight. To extract the peptides, a solution consisting of 50% (v/v) acetonitrile in 5% (v/v) formic acid and 80% (v/v) in 5% (v/v) formic acid was employed.

### Mass spectrometry analysis

The peptide samples extracted through in-gel digestion were resuspended in 20 µl of solvent A, which consisted of 0.1% formic acid prepared in water (Optima LC/MS grade, ThermoFisher Scientific). The separation of peptides was carried out using a PepMapTM RSLC C18 column (Thermo Fisher Scientific, San Jose, CA) with a linear gradient of Solvent B (0.1% FA in acetonitrile) ranging from 2% to 38% over a period of 65 min, and the flow rate was maintained at 300 nl/min. For analysis, the sample was introduced into an Orbitrap Fusion Lumos Tribrid mass spectrometer (Thermo Fisher Scientific, San Jose, CA), coupled with an Easy nanoLC 1000 system (Thermo Fisher Scientific, San Jose, CA). The spray voltage was set to 1.8 kV, and the heated capillary temperature was maintained at 275 °C. The instrument operated in a data-dependent mode, initiating with one full MS scan followed by twenty MS/MS scans, with a dynamic exclusion time of 20 s. In MS/MS scans, peptide fragmentation was achieved using higher energy collision dissociation (HCD). If oxonium product ions (*m*/*z* 204.0867, 138.0545) were detected in the HCD spectra, the peptides underwent further fragmentation using electron transfer/higher energy collision dissociation (EThcD) in a subsequent scan on the same precursor ion selected for HCD. Full MS scans were acquired in the range of 300 to 1400 m/z, with the first mass for HCD and EThcD MS/MS scans set at 120 and 110 m/z, respectively. The resolutions for full MS scans and MS/MS scans (HCD and EThcD) were 120,000 and 300,000, respectively. In full scans, the advanced gain control target was set at 4 × 105, with a maximum injection time of 100 ms. In MS/MS scans, the advanced gain control target was set at 5 × 104, with a maximum injection time of 54 ms, and the isolation window was set at 1.6 *m*/*z*. For EThcD MS/MS scans, the advanced gain control target was set at 8.5 × 104, with a maximum injection time of 54 ms, and an isolation window of 1.6 *m*/*z*. The HCD collision energy was maintained at 28%, and the ETD reaction time was 50 ms, with a supplemental activation (SA) collision energy of 20%.

The raw data underwent comparison with the Uniprot human database (release entry no. 173,324) using the SEQUEST HT search engine within Proteome Discoverer 2.2 (Thermo Fisher Scientific). For the proteolytic enzyme, trypsin was selected, allowing for a maximum of two missed cleavages. Precursor mass tolerance was set to 10 ppm and fragment mass tolerance was set to 0.02 Da. In this search, the weights of b, y ions, as well as c and z ions, were all set to 1. Cysteine carbamidomethylation was set in a fixed modification, while variable modification settings included oxidation of methionine and O-GlcNAcylation of serine and threonine, with a maximum of three post-translational modifications (PTMs) allowed on a single peptide. The minimum peptide length was set to six amino acids, and there was a requirement of at least one peptide per protein. The maximum delta Cn value at the fixed value PSM validator node was established at 0.05. IMP-ptm RS node was used with report only PTM, use diagnostic ions and use fragment mass tolerance of search node. Peptides and proteins were identified with a confidence level of ≥95%.

### Quantitative RT-PCR analysis

Total RNA was isolated using Trizol and subjected to reverse transcription (RT) using ReverTra Ace qPCR RT Master Mix (Toyobo, #FSQ-201). Quantitative RT-PCR was performed using TB Green Premix Ex Taq II (Takara, #RR820) with 200 nM of the primer sets listed in Supplementary Table 1 and 100 ng of cDNA per reaction. Reactions were performed on a CFX Duet real-time PCR system (Bio-rad). *beta-actin* was used as housekeeping gene control and a standard 2^-ΔΔCt^ analysis was performed.

### RNA sequencing analysis

For quality checks and read trimming, RNA-seq data were processed by FastQC (version 0.11.8) [36] and sickle (version 1.33) [37] with default parameters. After the trimming, the reads were aligned to human transcriptomes (GENCODE version 34, GRCH37/hg19) [38] using STAR (version 2.5.3a_modified) [39] with default parameters and read counts were determined using RSEM (version 1.3.1) [40]. To discover differentially expressed genes (DEGs), we utilized edgeR R package (version 3.32.1) [41] with batch information added as confounding variables to adjust for batch effects. To search for enriched gene ontology (GO) terms in set of DEGs (log2 fold change $\ge$ 2 and FDR $\le$ 0.05), we utilized g:Profiler, which is a web server for functional enrichment analysis of gene lists [42]. Gene set enrichment analysis (GSEA) was conducted using gseapy (version 1.0.5) [43].

### Cell viability assay

Cells were plated in 96-well plates at a density of 1 × 10^3^ cells per well. At specified time intervals following cell attachment, 10 μL of Quanti-Max WST-8 cell viability assay solution (Biomax, #QM1000, Korea) was introduced to each well, and the cells were incubated for 2 h at 37 °C. Subsequently, the absorbance was measured using a Multiskan Sky microplate spectrophotometer (Thermo Fisher Scientific) at a wavelength of 450 nm.

### Transwell invasion assay

Invasion assays were conducted utilizing Corning Matrigel Matrix (Corning, USA) and a 24 mm Transwell with a 0.8 μm insert, following the guidelines provided by the manufacturer. Following a 4-day incubation period, the quantification of invasive cells was performed using the Quanti-Max WST-8 cell viability assay solution (Biomax, #QM1000, Korea).

### Soft agar colony formation assay

The CytoSelect 96-well Cell Transformation Assay (Cell Biolabs, USA) was employed for the colony formation assay, according to the manufacturer’s protocol. In brief, cells were seeded in soft agar at a concentration of 1 × 10^4^ cells per well. After incubation at 37 °C in 5% CO_2_ for 1 and 14 days, the soft agar was solubilized, and cells were lysed using lysis buffer. The resulting cell lysates were incubated with the CyQUANT GR Dye and the readings were taken at the 485/520 nm filter set.

### Xenograft mouse experiment

For the xenograft mouse experiment, NONO knockout, reconstituted with WT, and reconstituted with T440A HCT116 cells were harvested using trypsin solution and washed with PBS. The cells were then suspended at a concentration of 1*10^7^ per 100 μL of PBS and injected into randomly divided three groups of 5-week-old male BALB/c nude mice obtained from DBL (*n* = 6 per group). Tumor size and weight were assessed two months post-injection.

### Quantifications

All data were analyzed as the mean ± standard deviation. Statistical analyses were performed using unpaired two-tailed *t*-test. One-way Analysis of Variance (ANOVA) with Tukey’s multiple comparisons test was used to compute statistical significance between multiple groups. GraphPad Prism software (Ver. 9) was used to determine statistical significance among multiple studies. Statistical significance was considered at *P < 0.05; **P < 0.01; ***P < 0.001.

## Supplementary information

Supplementary figure legends

Figure S1

Figure S2

Figure S3

Figure S4

Figure S5

Figure S6

Figure S7

Original Blots

Supplementary Table 1

Dataset S1

Dataset S2

Dataset S3

## Data Availability

All data reported in this paper will be shared by the lead contact upon request.

## Code availability

This paper does not report original code. Raw RNA-seq reads have been deposited in the NCBI Gene Expression Omnibus (GEO) database under accession codes, GSE262460 and Korea Sequence Read Archive (KRA) under accession codes, KAP240707. Any additional information required to reanalyze the data reported in this paper is available from the lead contact upon request.
