# Supplementary material for: O-GlcNAcylation of NONO regulates paraspeckle component assembly and contributes to colon cancer cell proliferation
Source: Cell Death Discov. 2025 May 13;11:234. doi: 10.1038/s41420-025-02405-z (PMC12075841; doi:10.1038/s41420-025-02405-z)
Supplement: Supplementary file 1 — Supplementary figure legends [file 41420_2025_2405_MOESM1_ESM.docx]

**Supplementary Information**

***O*-GlcNAcylation of NONO is Critical for Paraspeckle Component Assembly and Contributes to Colon Cancer Cell Proliferation**

Yeolhoe Kim^1,5^, Kyung-Tae Lee^2,5^, Han Byeol Kim^3^, Hyeryeon Jung^3^, Jeong Yeon Ko^1^, Tae Hyun Kweon^1^, Hari Chandana Yadavalli^1^, Junghwa Seo^4^, Suena Ji^4^, Yun Ju Kim^1^, Donghyuk Shin^1^, Seong Wook Yang^1^, Myeong Min Lee^1^, Jin Won Cho^4^, Eugene C. Yi^3^, Jin-Wu Nam*^2^, Won Ho Yang*^1,4,6^

^1^Department of Systems Biology, College of Life Science and Biotechnology, Yonsei University, 50 Yonsei-ro, Seodaemun-gu, Seoul 03722, Republic of Korea

^2^Department of Life Sciences, College of Natural Science, Hanyang University, Seoul 04736, Republic of Korea

^3^Department of Molecular Medicine and Biopharmaceutical Sciences, School of Convergence Science and Technology and College of Medicine or College of Pharmacy, Seoul National University, 28 Yeongeon-dong, Jongno-gu, Seoul 03080, Republic of Korea

^4^Glycosylation Network Research Center, Yonsei University, 50 Yonsei-ro, Seodaemun-gu, Seoul 03722, Republic of Korea

^5^These authors contributed equally

^6^Lead contact

*Corresponding authors:

bionicwono@yonsei.ac.kr, jwnam@hanyang.ac.kr

**Supplementary Figure legends**

**Figure S1. The number of NONO-containing paraspeckle-like structures in mouse liver tissue is regulated by *O*-GlcNAcylation status.**

**(A)** NONO immunostaining on liver cryosections in normal condition, 12 hr fasting, and combined 12 hr fasting with Thiamet-G IP injected mice.

**(B)** Cellular *O*-GlcNAcylation status was detected via Western blot.

Result in **(A)** was represented as boxes and whiskers: 10-90 percentile range, “+” sign represents mean, n > 50 nuclei from 3 different biologically independent replicates.

****P* < 0.001, one-way ANOVA with Tukey’s multiple comparisons test. Scale bar represents 5 μm.

**Figure S2. The number of NONO-containing paraspeckle-like structures in MCF7, A549 cells is regulated by *O*-GlcNAcylation status.**

**(A)** NONO immunostaining on breast cancer cell MCF7 in siControl, or siOGT conditions.

**(B)** Cellular *O*-GlcNAcylation status was detected via Western blot.

**(C)** NONO immunostaining on lung cancer cell A549 in siControl, or siOGT conditions.

**(D)** Cellular *O*-GlcNAcylation status was detected via Western blot.

**(E)** Relative NONO protein abundance in myc-OGT or control vector transfected HCT116 cell (*n*=3 per condition).

**(F)** Relative NONO protein abundance in V5-OGA or control vector transfected HCT116 cell (*n*=3 per condition).

**(G)** Relative NONO protein abundance in Thiamet-G or PBS-treated HCT116 cell (*n*=3 per condition).

**(H)** Relative NONO protein abundance in OSMI4 or DMSO-treated HCT116 cell (*n*=3 per condition).

Results in **(A, C)** are represented as boxes and whiskers: 10-90 percentile range, “+” sign represents mean, n > 50 nuclei from 3 different biologically independent replicates.

Data are presented as mean ± SD; ****P* < 0.001, Unpaired two-tailed *t*-test. Scale bar represents 5 μm.

**Figure S3. NONO protein is *O*-GlcNAcylated exogenously.**

**(A)** HEK293 cells were transfected with FLAG-tagged NONO constructs. Immunoprecipitation was performed with FLAG^®^-agarose.

**(B)** CBB stained gel for mass spectrometry analysis.

**Figure S4. *O*-GlcNAcylation level is responsible for paraspeckle complex formation in MCF7, A549 cells.**

**(A, B)** A co-IP assay was performed to evaluate the interactions between NONO with SFPQ, and PSPC1. MCF7 cells were treated with OSMI4 or siOGT and lysed after 24hrs or 48hrs, respectively. WCLs were subjected to IP with anti-NONO antibody. Relative co-immunoprecipitated SFPQ, PSPC1 level was normalized to NONO (*n*=3 per condition).

**(C, D)** A co-IP assay was performed to evaluate the interactions between NONO with SFPQ, and PSPC1. A549 cells were treated with OSMI4 or siOGT and lysed after 24hrs or 48hrs, respectively. WCLs were subjected to IP with anti-NONO antibody. Relative co-immunoprecipitated SFPQ, PSPC1 level was normalized to NONO (*n*=3 per condition).

Data are presented as mean ± SD; **P* < 0.05, ***P* < 0.01, ****P* < 0.001, Unpaired two-tailed *t*-test.

**Figure S5.** **Nuclear paraspeckle-like structure formation and the expression level of microtubule-related genes is dependent on NONO *O*-GlcNAcylation status.**

**(A**) NONO immunostaining on colon cancer cell HCT116 in siControl, or siSFPQ conditions.

**(B)** Western blot analysis for siControl or siSFPQ treated HCT116.

**(C)** qPCR was performed to evaluate the genes associated with microtubule cytoskeleton organization involved in mitosis (*n*=3 per condition).

**(D)** NONO immunostaining on NONO knockout HCT116 stably expressing T440A NONO in vector or myc-tagged SFPQ overexpressed conditions.

**(E)** Western blot analysis for vector or myc-tagged SFPQ overexpressed NONO knockout HCT116 stably expressing T440A NONO.

**(F)** qPCR was performed to evaluate the genes associated with microtubule cytoskeleton organization involved in mitosis (*n*=3 per condition).

**(G)** PSPC1 immunostaining on colon cancer cell HCT116 in siControl, siOGT or siOGT with FLAG-tagged WT NONO overexpressed conditions.

**(H)** Western blot analysis for siControl, siOGT or siOGT with FLAG-tagged NONO overexpressed HCT116.

**(I)** qPCR was performed to evaluate the genes associated with microtubule cytoskeleton organization involved in mitosis (*n*=3 per condition).

Results in **(A, D, G)** are represented as boxes and whiskers: 10-90 percentile range, “+” sign represents mean, n > 50 nuclei from 3 different biologically independent replicates.

Data are presented as mean ± SD; **P* < 0.05, ***P* < 0.01, ****P* < 0.001 (**(A, C, D, F)**; Unpaired two-tailed *t*-test, **(G, I)**; one-way ANOVA with Tukey’s multiple comparisons test)

**Figure S6. NONO protein level contributes to HCT116, MCF7, and A549 cell proliferation.**

**(A)** NONO expression was detected via Western blot analysis. HCT116 cells were treated with siControl or siNONO and lysed after 48 hrs. WST-8 assay was performed to compare cell growth between siControl or siNONO-treated HCT116 cells (*n*=3 per condition).

**(B)** NONO expression was detected via Western blot analysis. MCF7 cells were treated with siControl or siNONO and lysed after 48 hrs. WST-8 assay was performed to compare cell growth between siControl or siNONO-treated MCF7 cells (*n*=3 per condition).

**(C)** NONO expression was detected via Western blot analysis. A549 cells were treated with siControl or siNONO and lysed after 48 hrs. WST-8 assay was performed to compare cell growth between siControl or siNONO-treated A549 cells (*n*=3 per condition).

Data are presented as mean ± SD; ****P* < 0.001, Unpaired two-tailed *t*-test.

**Figure S7. Other DBHS proteins, SFPQ and PSPC1, were *O*-GlcNAcylated exogenously.**

**(A)** HEK293 cells were transfected with FLAG-tagged SFPQ constructs. Immunoprecipitation was performed with FLAG^®^-agarose.

**(B)** HEK293 cells were transfected with FLAG-tagged PSPC1 constructs. Immunoprecipitation was performed with FLAG^®^-agarose.
