# Supplementary figures and images for: O-GlcNAcylation of NONO regulates paraspeckle component assembly and contributes to colon cancer cell proliferation
Source: Cell Death Discov. 2025 May 13;11:234. doi: 10.1038/s41420-025-02405-z (PMC12075841; doi:10.1038/s41420-025-02405-z)

**A**

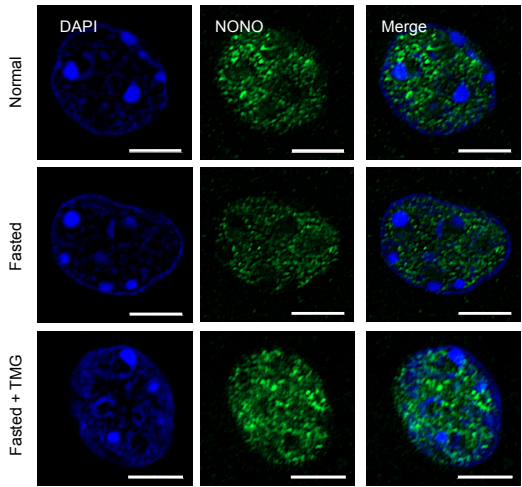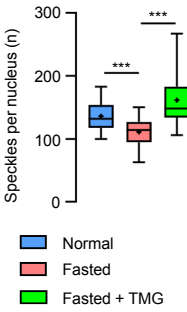

**B**

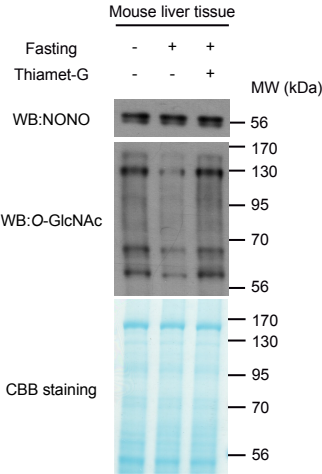

Supplement: Supplementary file 2 — Figure S1 [file 41420_2025_2405_MOESM2_ESM.pdf]

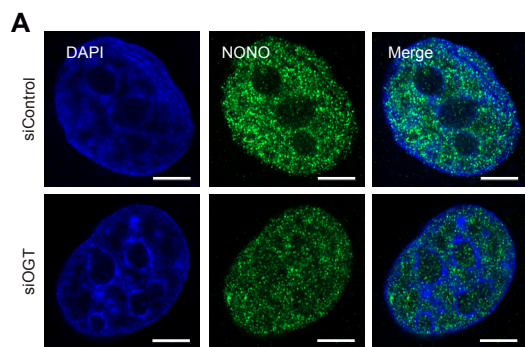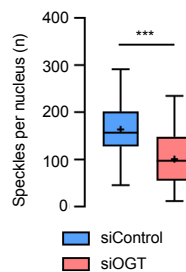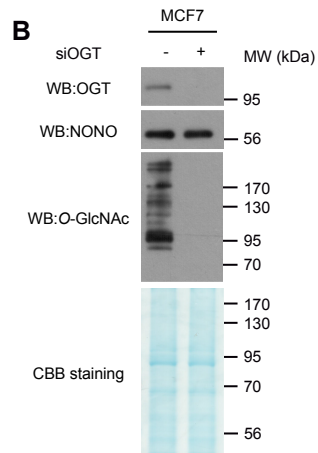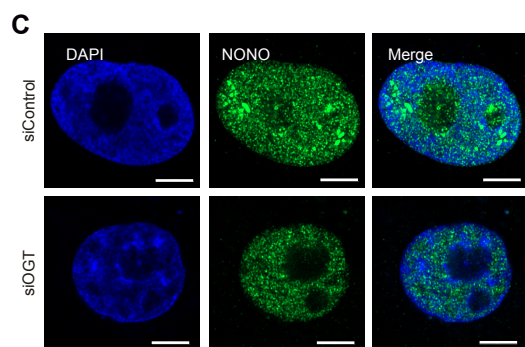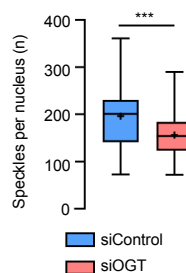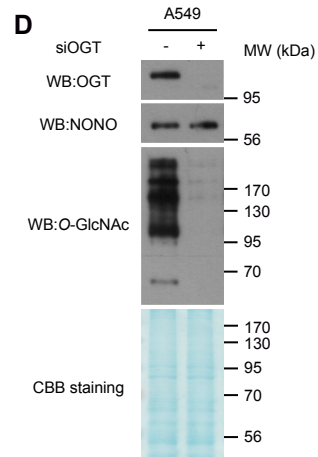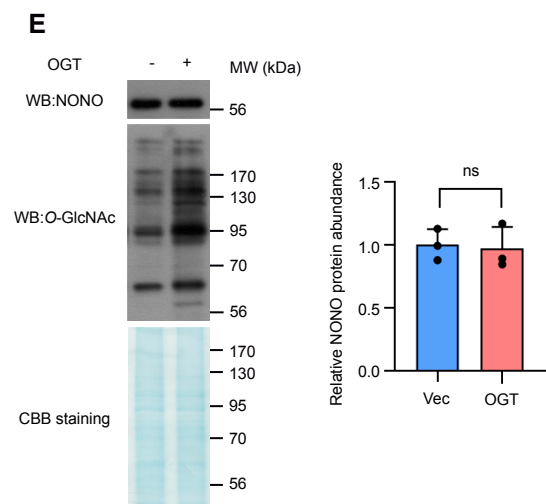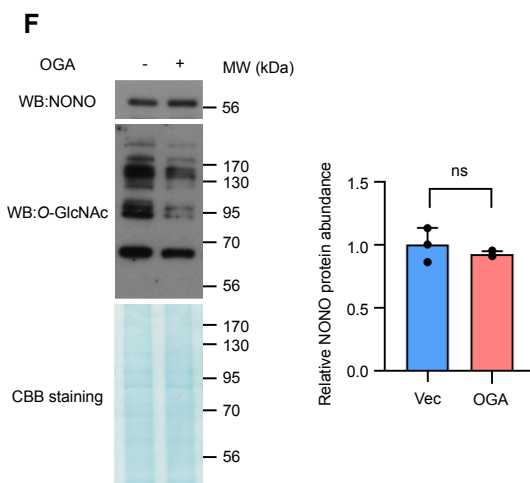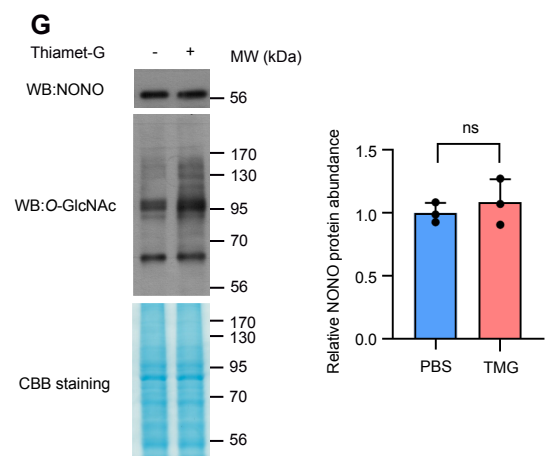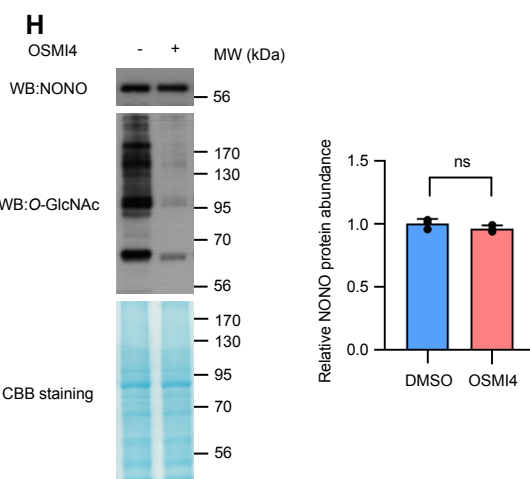

Supplement: Supplementary file 3 — Figure S2 [file 41420_2025_2405_MOESM3_ESM.pdf]

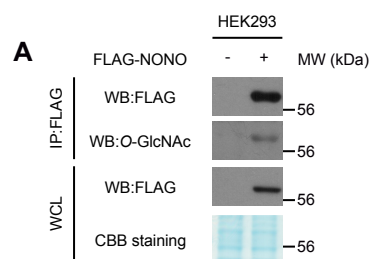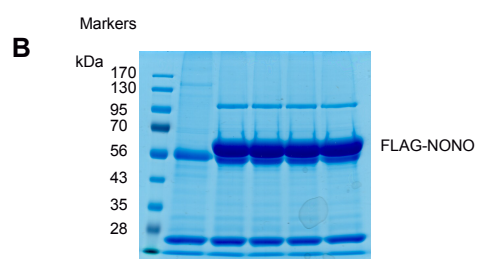

Supplement: Supplementary file 4 — Figure S3 [file 41420_2025_2405_MOESM4_ESM.pdf]

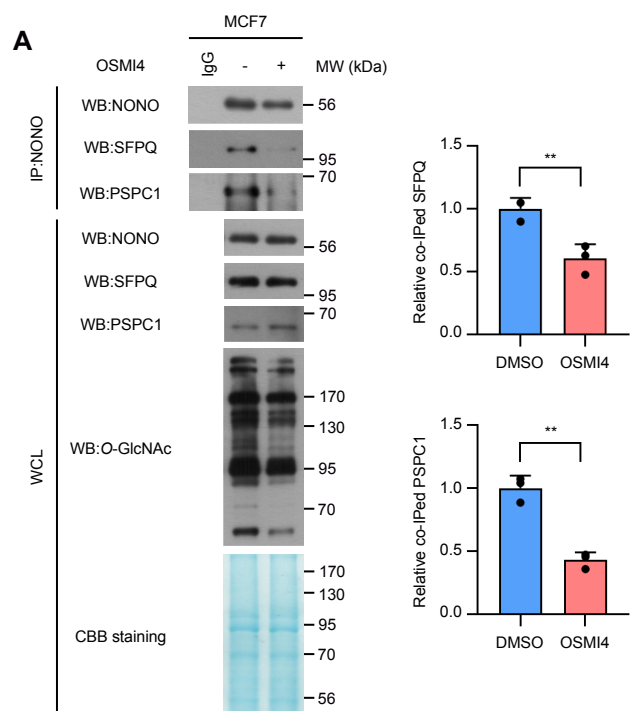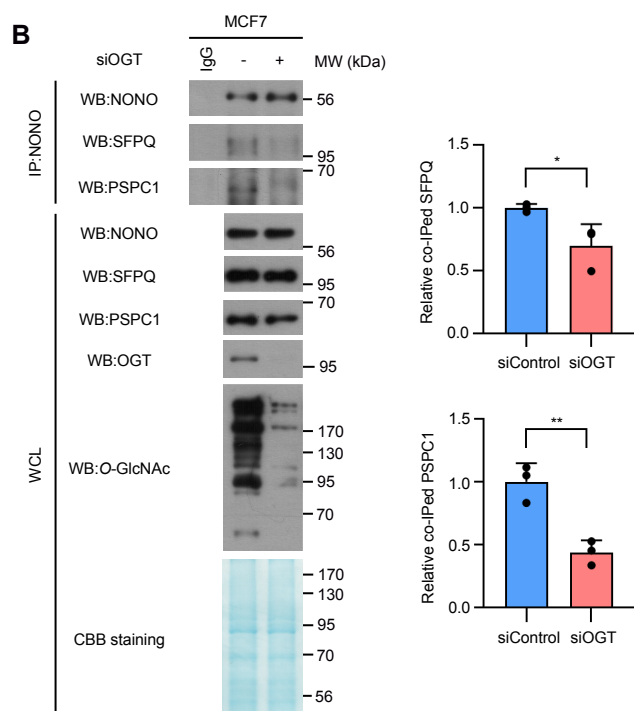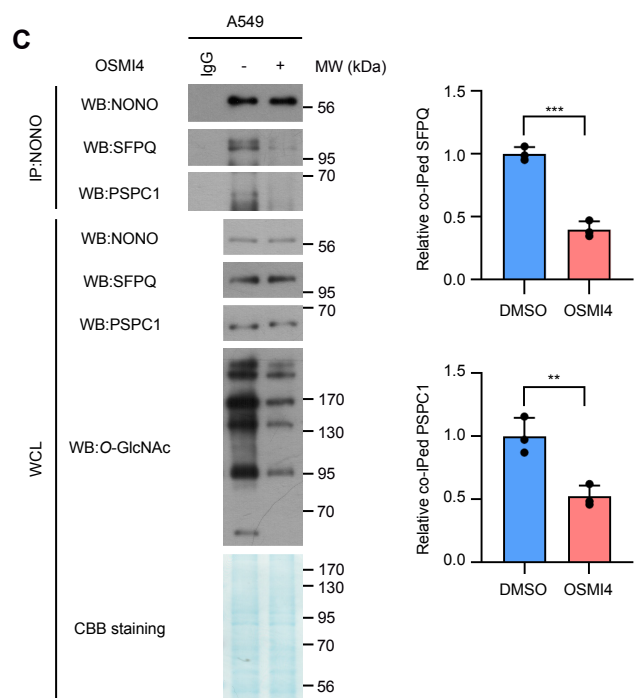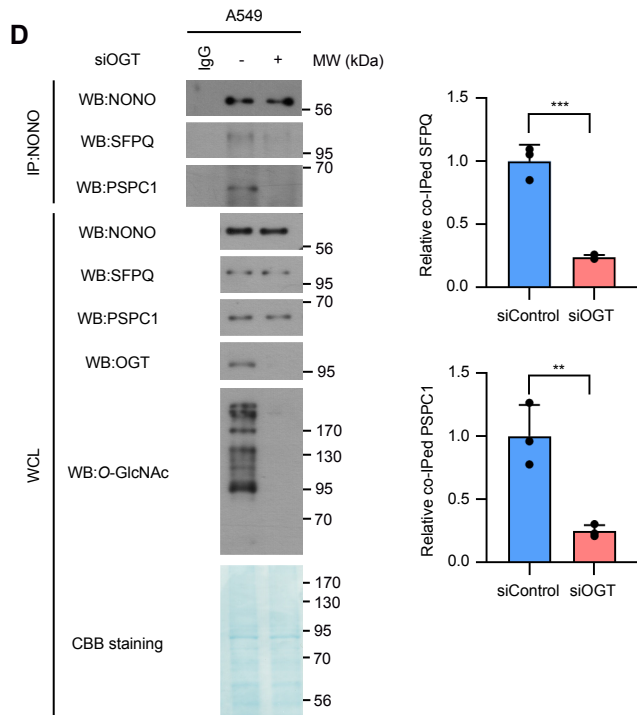

Supplement: Supplementary file 5 — Figure S4 [file 41420_2025_2405_MOESM5_ESM.pdf]

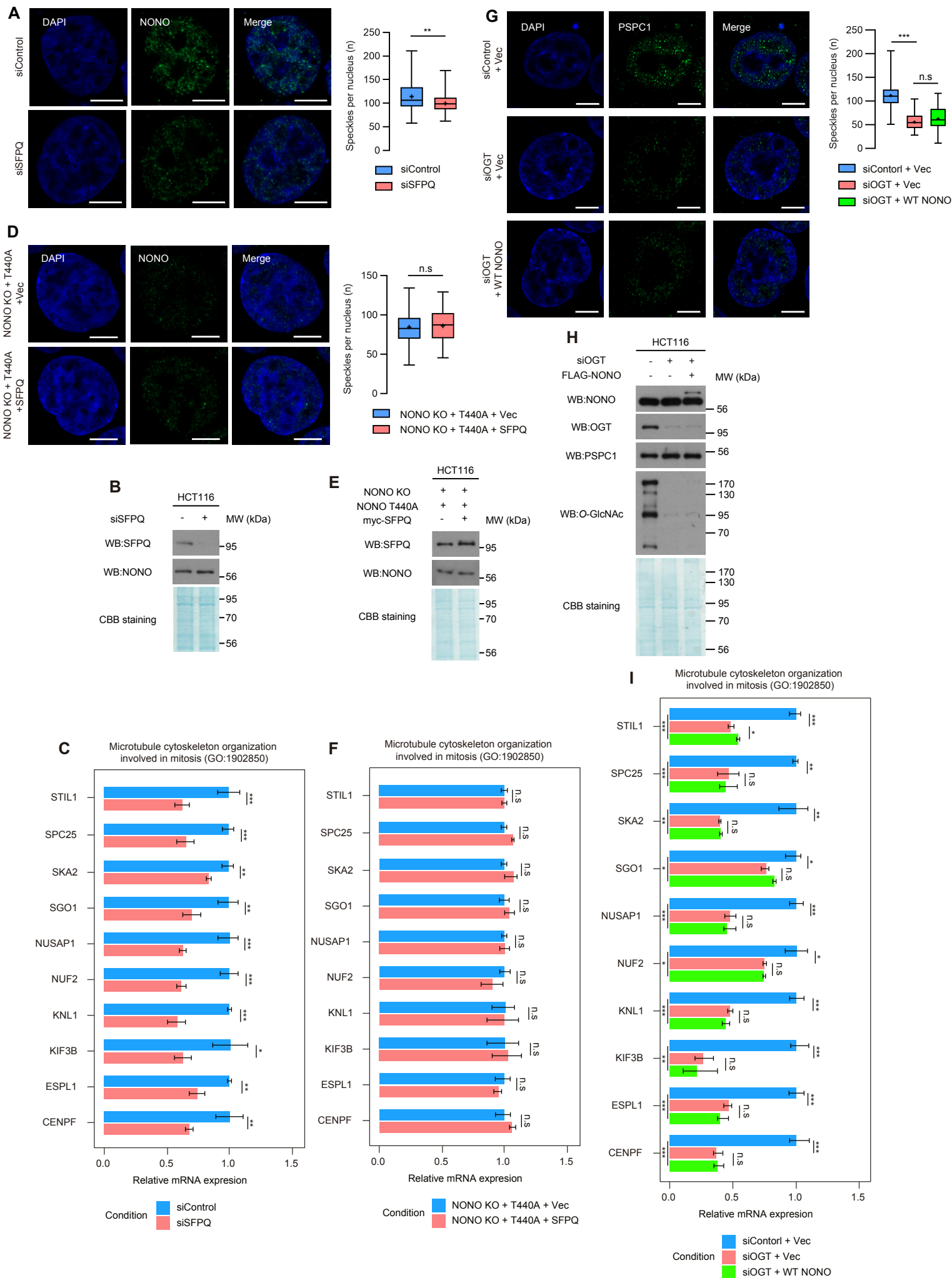

Supplement: Supplementary file 6 — Figure S5 [file 41420_2025_2405_MOESM6_ESM.pdf]

**A**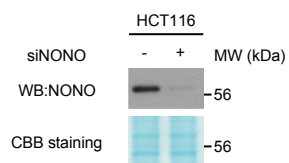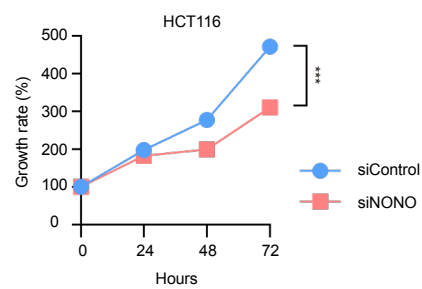**B**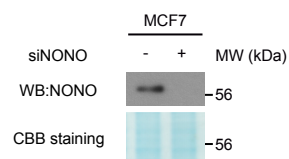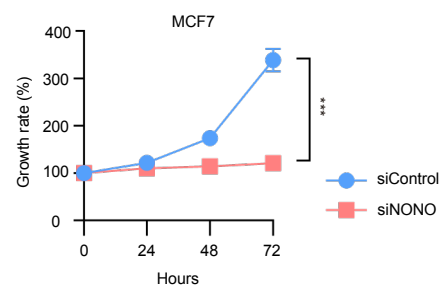**C**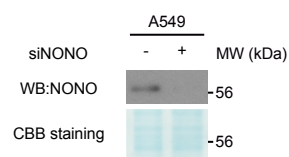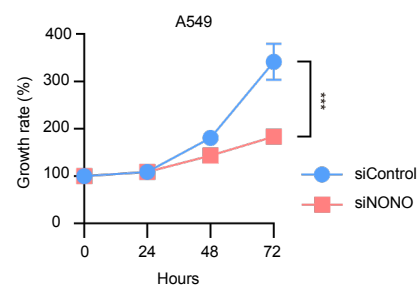

Supplement: Supplementary file 7 — Figure S6 [file 41420_2025_2405_MOESM7_ESM.pdf]

**A**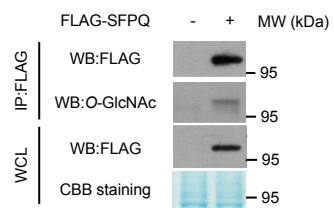**B**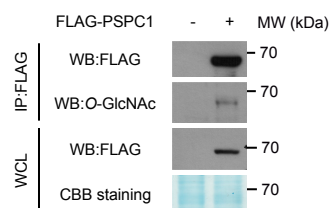

Supplement: Supplementary file 8 — Figure S7 [file 41420_2025_2405_MOESM8_ESM.pdf]
