# Supplementary material for: O-GlcNAcylation of NONO regulates paraspeckle component assembly and contributes to colon cancer cell proliferation
Source: Cell Death Discov. 2025 May 13;11:234. doi: 10.1038/s41420-025-02405-z (PMC12075841; doi:10.1038/s41420-025-02405-z)

Supplementary Material – Original Blots

Fig. 1

B

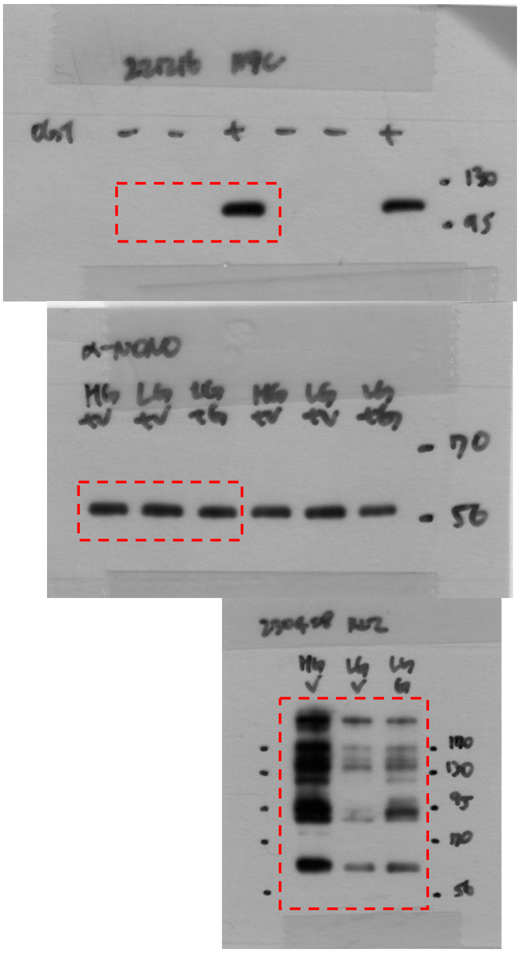

D

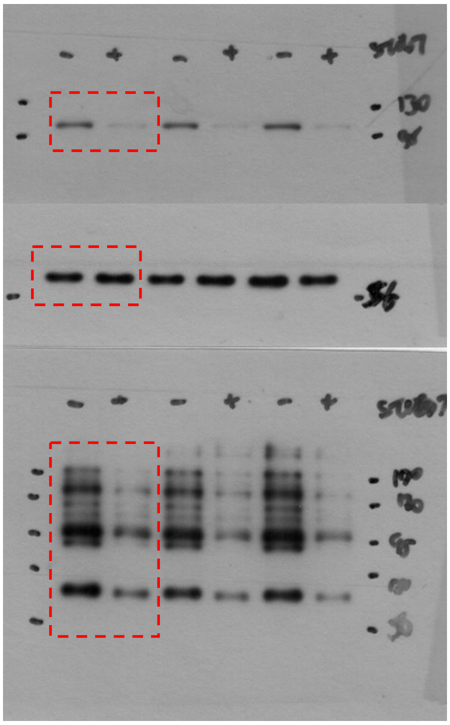

E

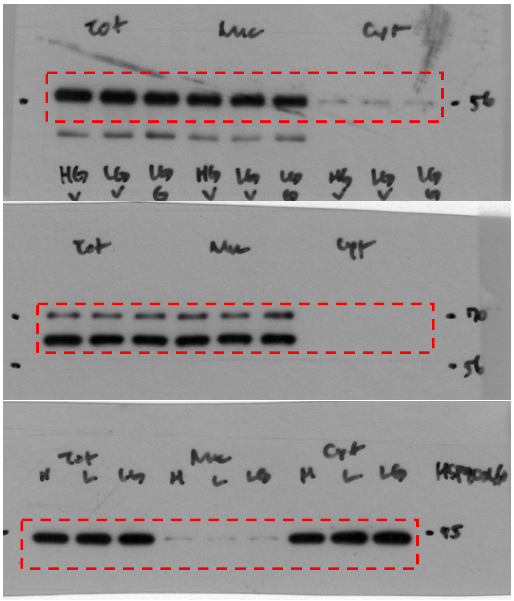

F

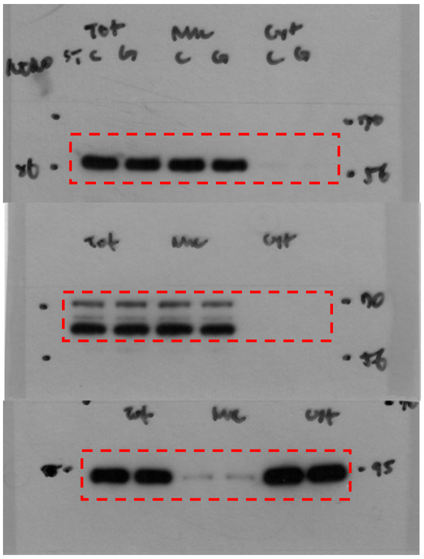

Fig. 2

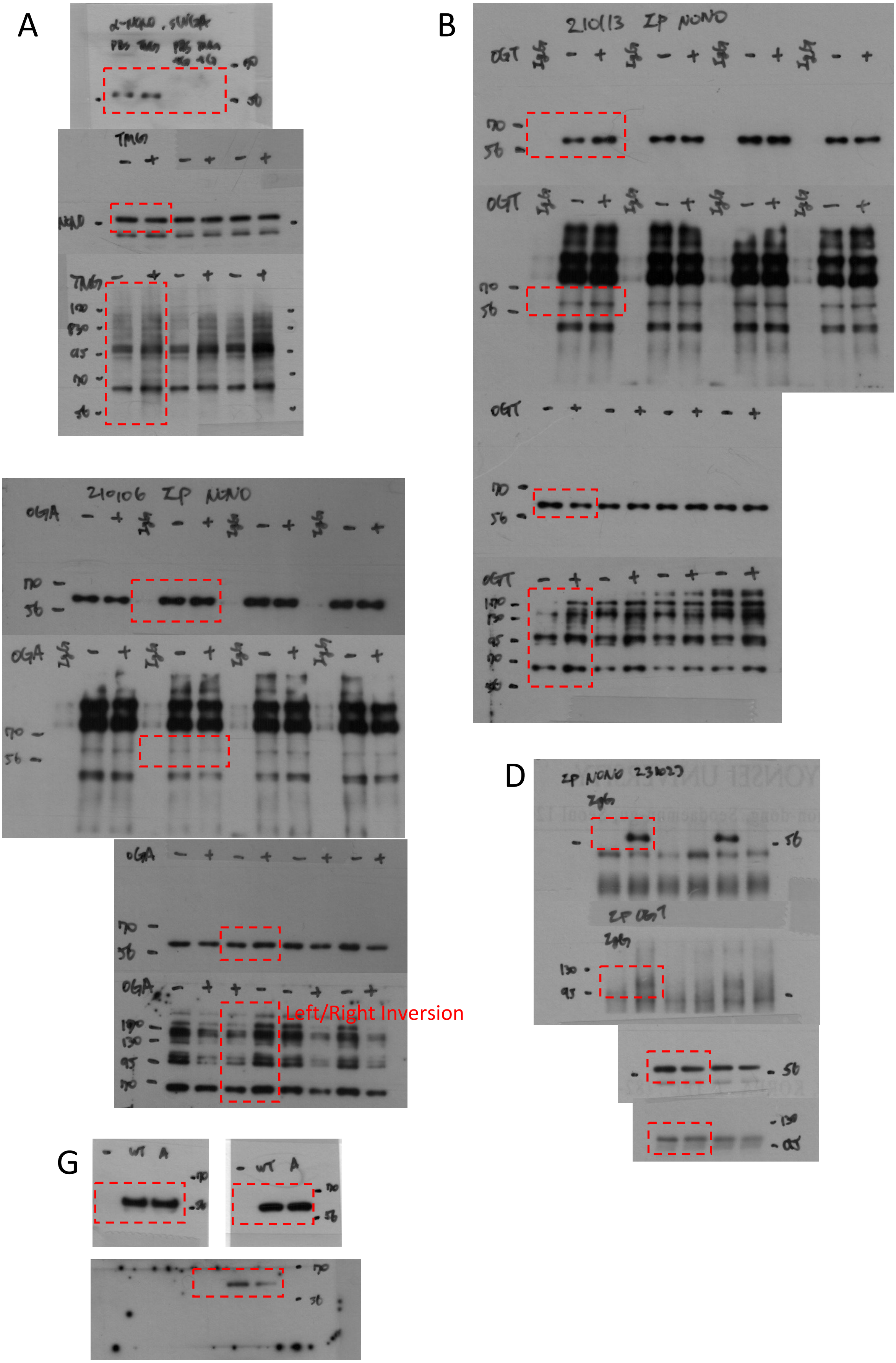

Fig. 3

A

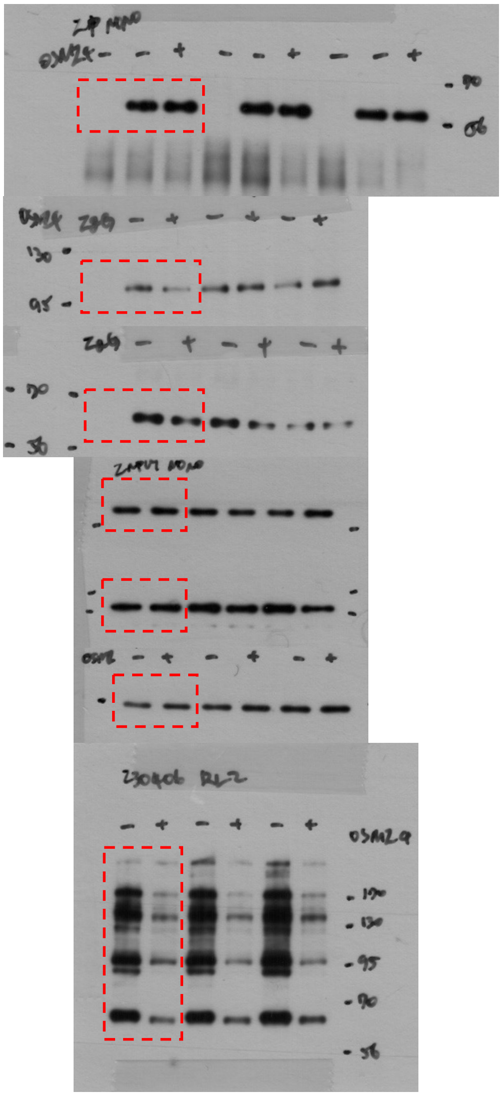

B

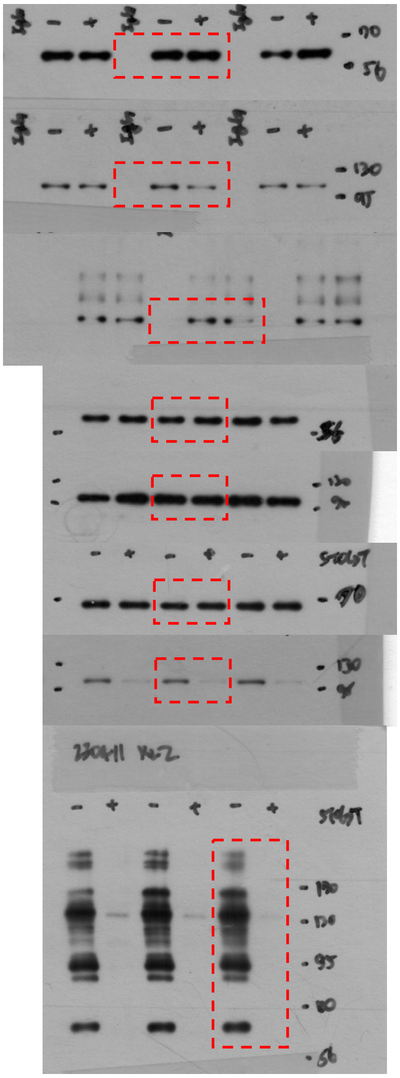

D

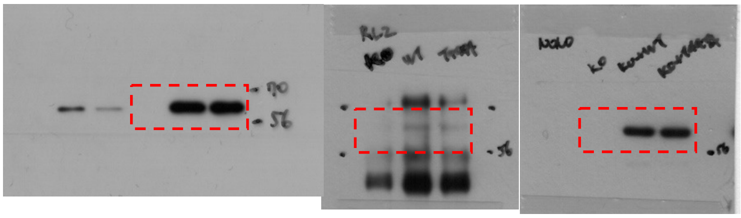

E

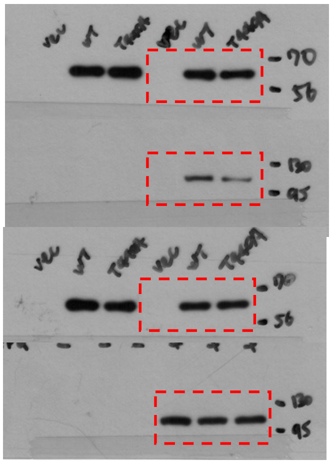

F

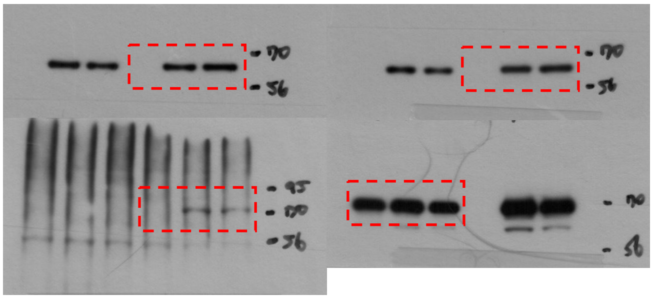

Fig. S1

B

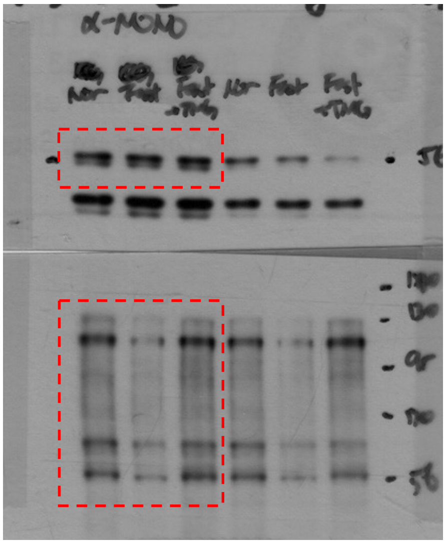

Fig. S2

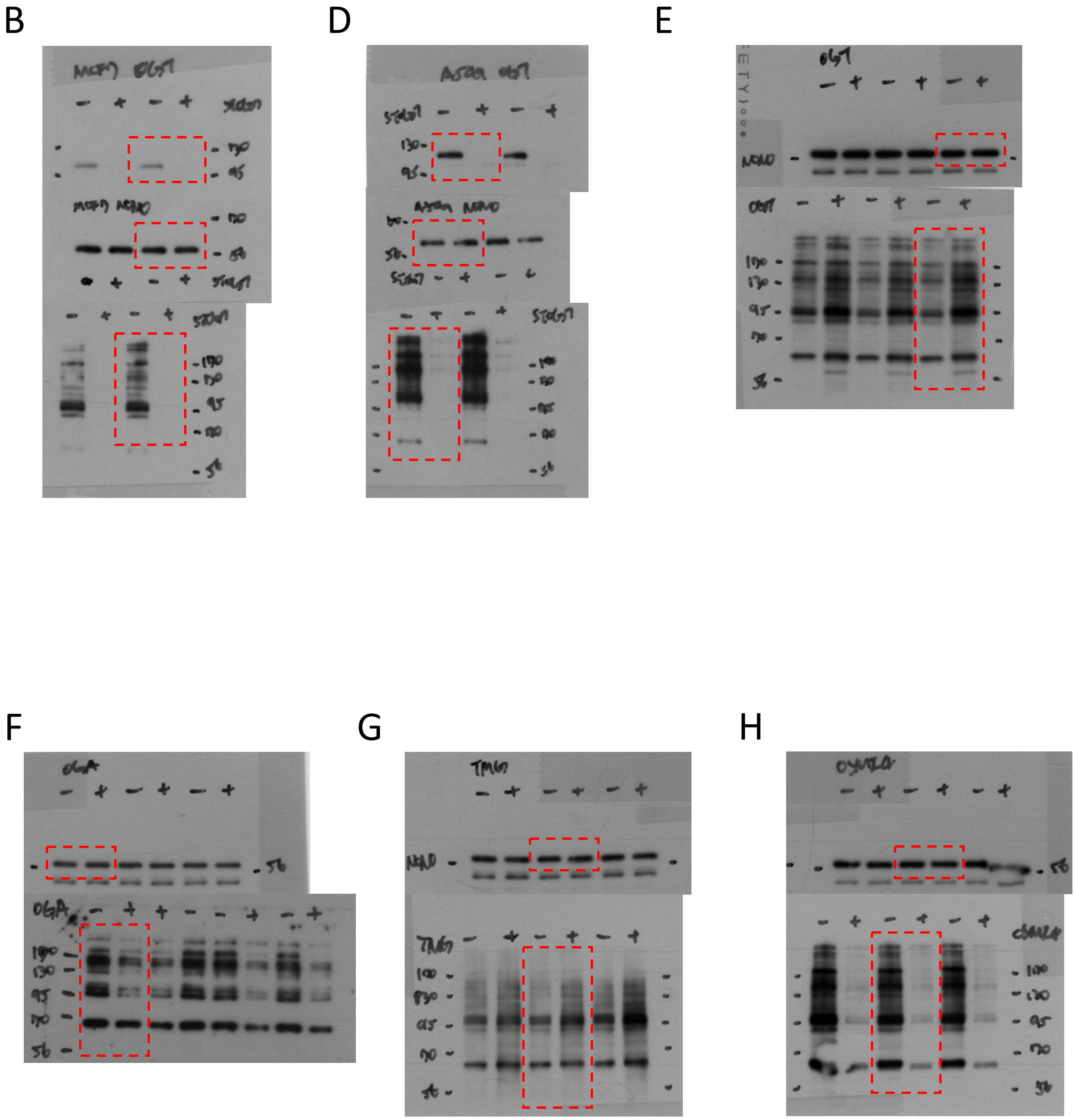

Fig. S3

A

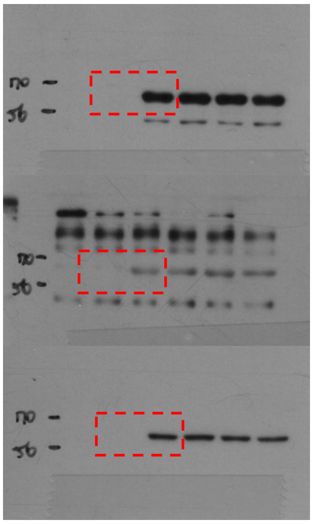

Fig. S3

A

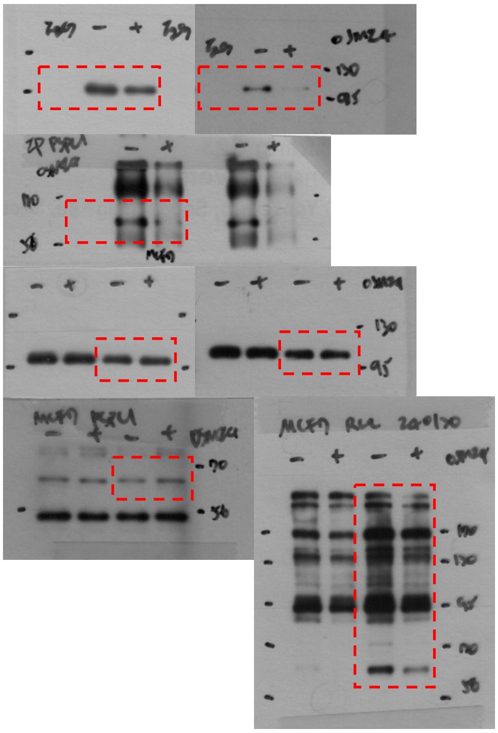

B

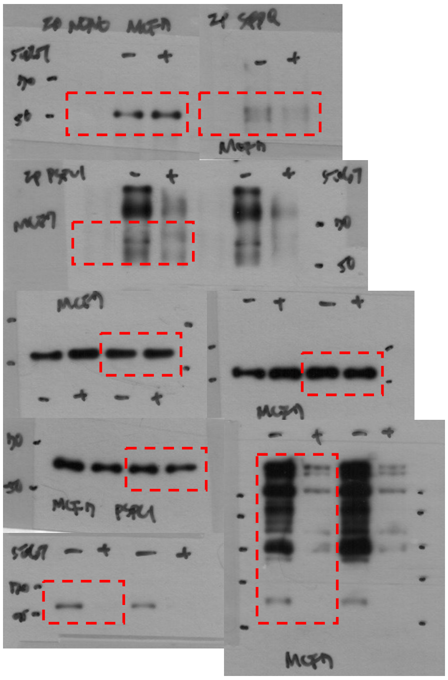

C

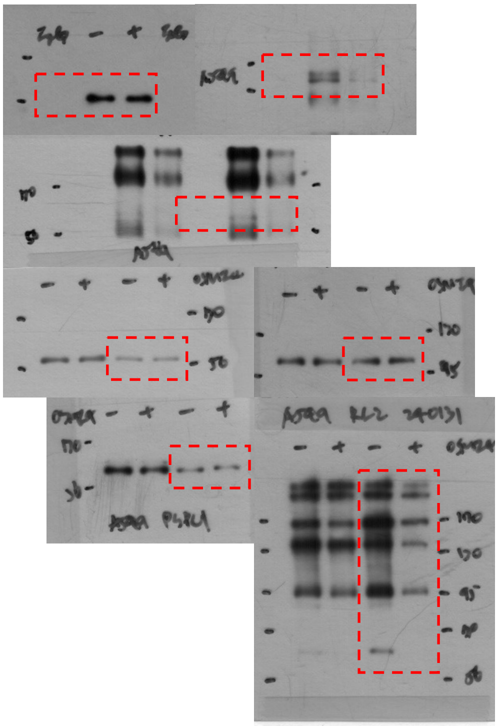

D

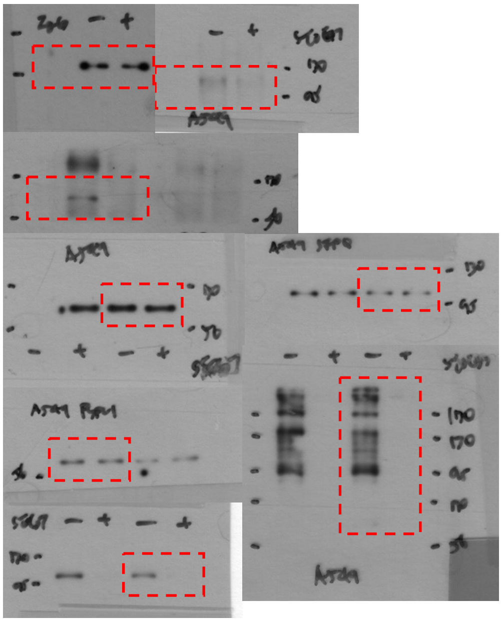

Fig. S5

B

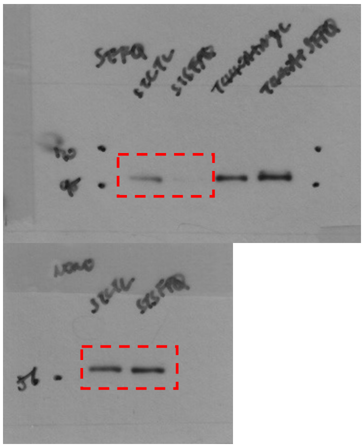

H

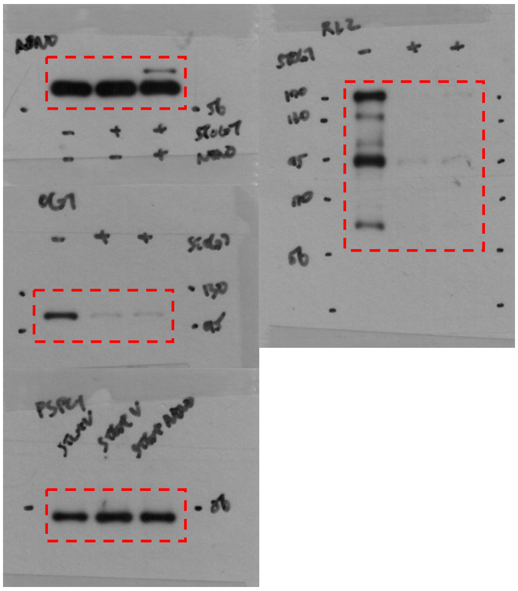

E

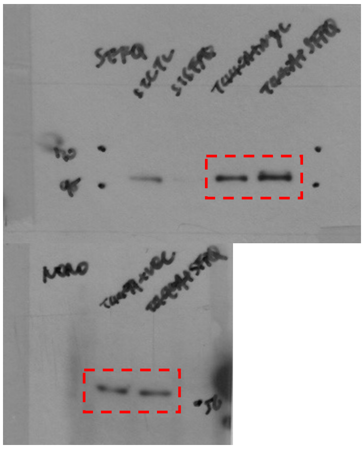

Fig. S6

A

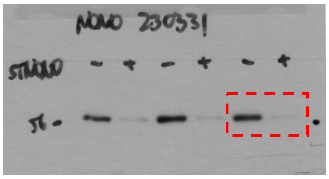

B

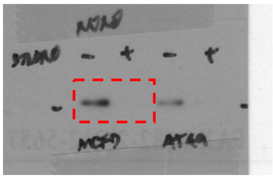

C

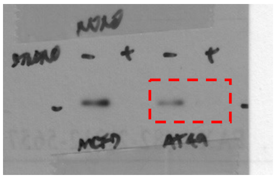

Fig. S7

A

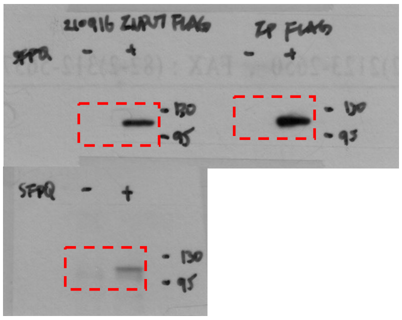

B

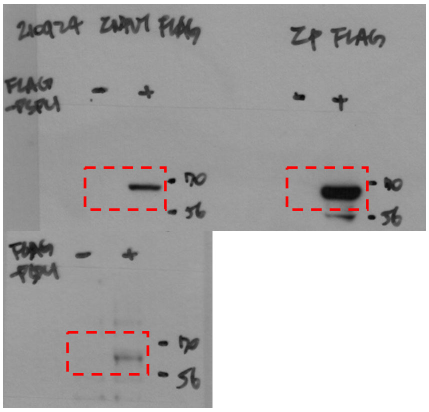

Supplement: Supplementary file 9 — Original Blots [file 41420_2025_2405_MOESM9_ESM.pdf]
